# Effects of the transtheoretical model-based self-management program on behavioral change in persons with epilepsy: Study protocol for a randomized controlled trial

**DOI:** 10.1371/journal.pone.0305547

**Published:** 2024-11-25

**Authors:** Cai Li, Junting Chen, Jin Tan, Ye Xiao, Renli Deng, Hao Huang

**Affiliations:** 1 Department of Neurology, Affiliated Hospital of Zunyi Medical University, Zunyi, Guizhou Province, China; 2 Nursing College of Zunyi Medical University, Zunyi City, Guizhou Province, China; 3 Good Clinical Practice Center, Affiliated Hospital of Zunyi Medical University, Zunyi City, Guizhou Province, China; University of the Witwatersrand Johannesburg, SOUTH AFRICA

## Abstract

**Introduction:**

Self-management applications are cost-effective and scalable for epilepsy treatment. However, there is a limited long-term quantitative evidence regarding their effectiveness. The Transtheoretical Model (TTM) offers a psychological framework that tailors self-management plans to the individual’s stage of behavioral change. This approach aims to address utilization needs, reduce information overload, minimize the adverse effects of self-management, and enhance the overall effectiveness of interventions for individuals with epilepsy.

**Methods and analysis:**

This will be a randomized, double-blind, the clinical trial involving two groups of adults diagnosed with epilepsy. In addition to treatment as usual (TAU), the intervention group will receive an intervention program constructed from TTM combined with self-management as part of the treatment plan. The control group will receive TAU only. Prior to the intervention, the participants will undergo an initial assessment to determine their current stage of behavioral change to facilitate the implementation of targeted behavior support strategies. Additionally, participants will receive weekly 30-minute educational videos on epilepsy. The goal is to enroll 160 adults who have been diagnosed with epilepsy for at least six months and are receiving medication. Data collection will encompass an assessment of C-ESMS, HADS, QOLIE-31, and LSSS. These parameters will be evaluated at baseline, as well as during follow-up periods at 1, 3, and 6 months post-intervention.

**Discussion:**

Existing self-management interventions for people with epilepsy primarily focus on knowledge and psychological factors. Discrepancies in research design, intervention plans, and patient characteristics have contributed to inconsistent outcomes in previous studies. This study seeks to advance the field by integrating the TTM with empirically supported self-management practices. The delineation of behavioral change stages within the TTM framework is expected to form a structured intervention protocol. This study will inform standardized, evidence-based epilepsy care practices.

## Introduction

Epilepsy, the second most burdensome neurological disorder worldwide, is characterized by abnormal neuronal discharges in the brain, leading to recurring seizures. Epilepsy affects approximately 70 million people globally [1], and it is associated with a high prevalence of comorbidities such as psychosis, depression, and cognitive dysfunction [2–5]. Furthermore, individuals with epilepsy are three times more likely to experience mortality from any cause compared with the general population [6]. Antiepileptic drug therapy is the linchpin of epilepsy management. Yet, the challenge of medication adherence cannot be overstated. Observational studies estimate that nearly 39% of patients with epilepsy exhibit less than optimal adherence to their prescribed drug regimens, a behavior linked to an increased incidence of recurrent seizures and a concomitant escalation in the annual rate of multiple seizure events [7–9]. In parallel, the phenomenon of pharmacoresistance, where an estimated 30% of adherent patients do not achieve satisfactory seizure suppression, presents a considerable clinical hurdle [7]. These factors highlight the complexity of epilepsy management. A dual-focused approach is necessary, addressing both adherence and individual pharmacological responsiveness to optimize therapeutic outcomes.

Despite the recognized importance of patient self-management education, healthcare providers often overlook this aspect due to time constraints, resource limitations, and competing clinical priorities. Out-of-hospital healthcare practitioners also face challenges in monitoring medication adherence, improving health behaviors, and coping with the skills needed for individuals with epilepsy. Notably, adults with epilepsy encounter numerous obstacles in managing their condition, such as a lack of comprehensive information about epilepsy, its treatment options, and potential consequences [8]. Additionally, individuals with epilepsy often experience anxiety and depression due to social stigma, high seizure frequency, job loss or dropout, and driving limitations [4, 9–11].

Self-management refers to the ways in which individuals with chronic illnesses handle various medical, role, and emotional issues [12]. In the context of epilepsy, self-management aims to equip individuals with the skills necessary to effectively manage their epilepsy and its associated effects. These skills include adherence to medication, accurate reporting and recording of seizure activity, implementation of safety precautions, and prioritizing adequate rest [13]. By introducing and encouraging self-management assistance for patients, individuals can cultivate confidence in their ability to treat and manage their condition. Moreover, self-management empowers individuals to develop invaluable skills that contribute to controlling and coping with their sickness, ultimately minimizing its impact on health and quality of life. This approach has the potential to bridge the gaps in care created by out-of-hospital treatment [14, 15]. While the Managing Epilepsy Well (MEW) Network has shown the effectiveness and accessibility of some epilepsy self-management-relevant research, it also offers potential benefits in terms of health care and healthcare service utilization [16–19]. However, the implementation of self-management strategies can be hindered by factors such as low literacy levels, limited health literacy, and cognitive differences among patients. These factors add complexity to self-management programs and often result in poorer outcomes [13, 20]. During the outreach phase of self-management interventions, Velicer et al. [21] observed that patients experienced diverse stages of behavioral change after adopting self-management strategies. Recognizing this, it is crucial for health promotion efforts to take into account an individual’s specific stage of behavioral change to enhance their effectiveness.

The Transtheoretical Model (TTM) is a comprehensive, dynamic model that aims to induce behavioral change through five stages: (1) pre-contemplation (no intention of taking action); (2) contemplation (intention of taking action within the next six months); (3) preparation (intention of taking action within the next 30 days); (4) action (behavioral change made within six months); and (5) maintenance (behavioral change sustained for six months or longer) [22]. The TTM incorporates constructs such as decision balance, self-efficacy, and change processes, which systematically correspond to different stages in a predictable manner [23]. The Transtheoretical Model (TTM) is increasingly acknowledged as a potent framework for catalyzing behavioral change in health-related behaviors [24–26]. A empirical evidence suggests that interventions predicated on TTM’s stage-specific principles are more efficacious than homogeneous interventions that fail to consider an individual’s stage of readiness for change [27]. This distinction is critical, as TTM-guided interventions are designed to be congruent with the individual’s current motivational stage, thereby offering a personalized and dynamic approach to behavior modification. In contrast, uniform interventions are typically static and may not sufficiently address the nuanced progression of behavioral change.

However, research on applying the TTM to epilepsy self-management interventions remains limited. This study aims to utilize the TTM and incorporate patients’ preferences and goals for a patient-centered approach to self-management practices. Lewinski et al. [28] suggested that understanding epilepsy-specific considerations and their impact on self-management can positively influence engagement and treatment adherence in self-management programs. However, the complexity of epilepsy management poses challenges to the application of TTM strategies. It is important to note that managing the effects of multiple behaviors on epilepsy and tailoring interventions to individuals’ willingness to change can present additional challenges for healthcare professionals. Nevertheless, these challenges also present opportunities that have yet to be explored.

## Materials and methods

### Study design and setting

**Fig 1:**
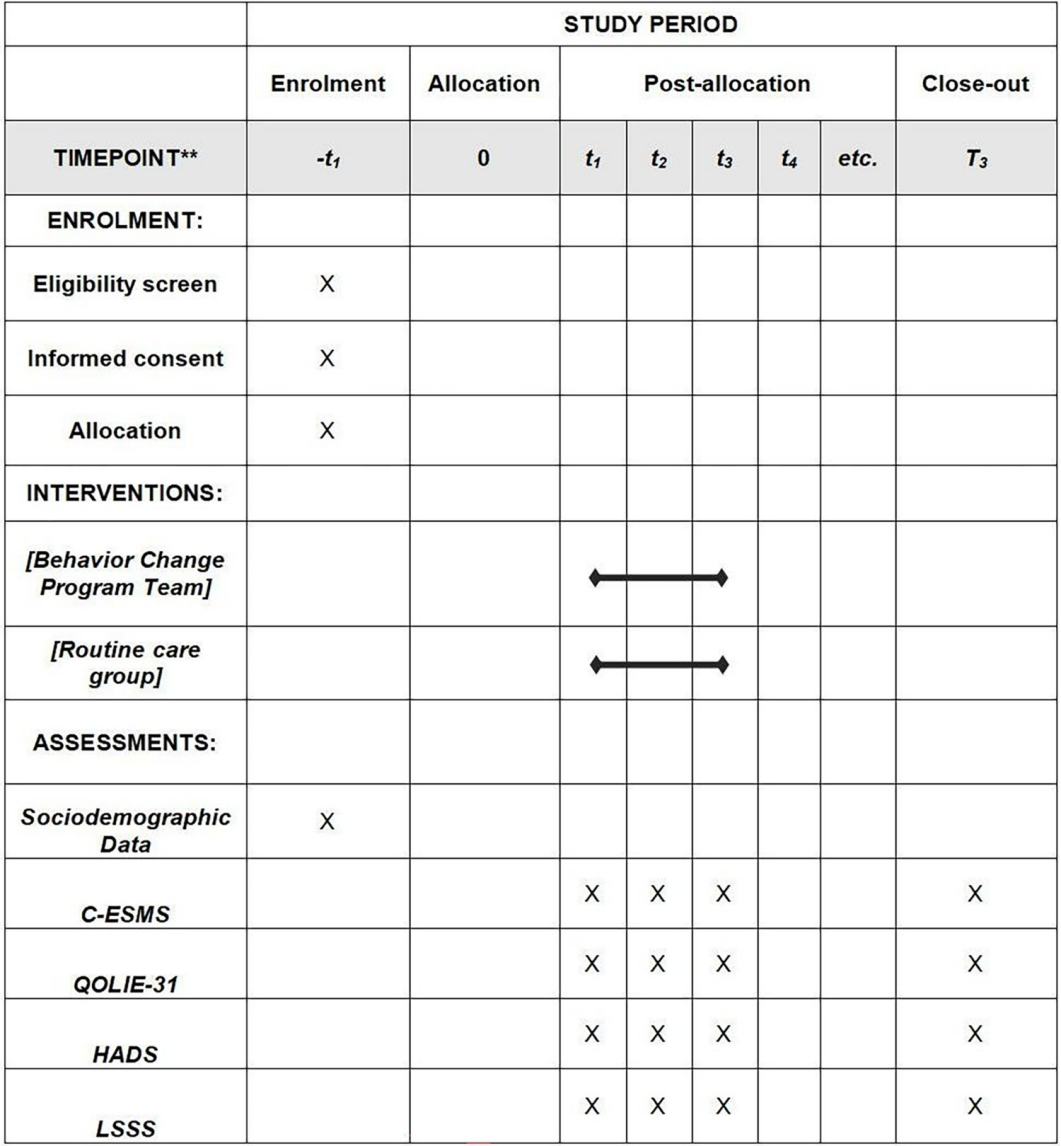
Schedule of enrolment, interventions, and assessments for epilepsy management study.

We will conduct a randomized clinical trial in accordance with the CONSORT recommendations, using two parallel arms and a double blind method. This trial is set to recruit participants and implement intervention at the Affiliated Hospital of Zunyi Medical University in southwest China from August 31, 2023 to April 30, 2024. The main purpose of this study is to evaluate the effectiveness of intervention measures in improving self-management behavior, health-related quality of life, anxiety and depression status, and seizure frequency in people with epilepsy. In addition, qualitative analysis will be conducted to explore the experience of the intervention group participating in the experiment, providing valuable insights for future interventions. This protocol adheres to the SPIRIT Checklist (S1 Checklist) [29] and has been registered with the Chinese Clinical Trial Registry under the number ChiCTR2300074975. A detailed schedule of enrollment, interventions, and assessments is provided in Fig 1. Should there be any significant methodological changes post-initiation, such as adjustments to eligibility criteria or interventions, we will document and justify these changes to ensure trial transparency and the integrity of outcomes. All amendments will be promptly updated in the trial registry and communicated to all relevant parties, maintaining compliance with ethical and regulatory standards.

### Participants and recruitment

**Fig 2:**
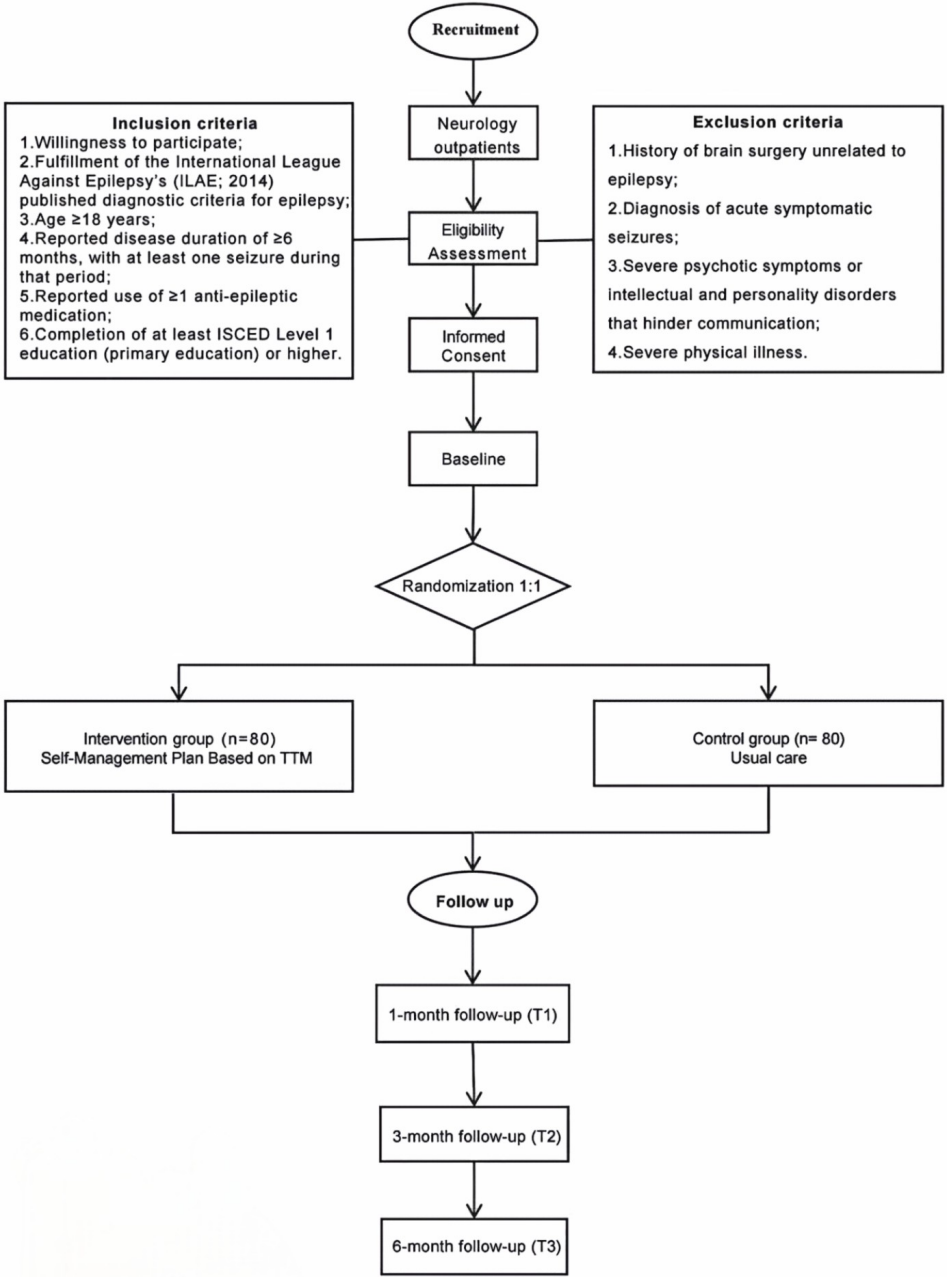
Recruitment process flowchart. (A)Ovals: Process start/end. (B)Rectangles:Steps. (C)Diamonds:Decisions. (D)Arrows:Flow.direction. (E)Lines:Connections between steps.

The proposed study is a randomized controlled trial to be conducted within the Neurology Department of the Affiliated Hospital of Zunyi Medical College, located in Southwest China. This department annually serves in excess of 70,000 outpatients and manages a long-term follow-up cohort that includes over 1,300 individuals diagnosed with epilepsy. Epilepsy specialists in the Neurology Department will systematically identify potentially eligible patients based on pre-determined inclusion criteria. This process will involve reviewing medical records, consulting with other healthcare professionals, and utilizing electronic medical databases. All patient information will be handled in accordance with ethical guidelines and local data protection regulations to ensure confidentiality and privacy. Subsequently, expert medical staff will provide brief information materials about the study to each potentially eligible patient. Patients who meet the inclusion criteria will then be introduced to neurology evaluators who will provide comprehensive explanations of the study, including its objectives, procedures, potential risks, and benefits of participation. These evaluators will address any concerns or questions raised by the patients and ensure their full understanding of the study. Once written informed consent is obtained (see the template for the consent form in S2 File), a baseline visit will be conducted by an assigned evaluator at the outpatient clinic. Randomization will then take place to assign patients to either the control group or the intervention group (see Fig 2 and the randomization section).

### Inclusion criteria

1. Willingness to participate;
2. Fulfillment of the International League Against Epilepsy’s (ILAE; 2014) published diagnostic criteria for epilepsy [30];
3. Age ≥18 years;
4. Reported disease duration of ≥6 months, with at least one seizure during that period;
5. Reported use of ≥1 anti-epileptic medication;
6. Completion of at least ISCED Level 1 education (primary education) or higher.

### Exclusion criteria

1. History of brain surgery unrelated to epilepsy;
2. Diagnosis of acute symptomatic seizures;
3. Severe psychotic symptoms (e.g.hallucinations) or intellectual and personality disorders that hinder communication;
4. Severe physical illness.

### Intervention

#### Intervention group

The intervention team will consist of a Chinese Medical Association (CMA) committee-certified neurologist and two experienced research nurses who will receive two days of professional training specifically for the intervention. This training will enhance their scholarship and skills in implementing behavioral change plans. In addition to receiving treatment as usual (TAU), the participants assigned to the intervention group will engage in a behavioral change plan, which will include individual one-hour-weekly sessions with the neurologist and research nurses for a duration of eight weeks. These sessions will involve educational lectures and PowerPoint presentations on various epilepsy-related topics, including medication management, identification of triggering factors, and seizure management. The sessions will be interactive, allowing the participants to ask questions and actively engage in discussions to enhance their understanding and self-management skills. Following the completion of the behavioral change intervention, the participants will receive monthly follow-up calls. These calls, which will last about 20 minutes each, will provide ongoing support, address any concerns, and monitor the participants’ progress in applying the self-management strategies learned during the intervention. If necessary, the intervention will be adjusted or modified based on individual circumstances and participant needs.

The study nurse will establish a collaborative therapeutic relationship with each participant. After a comprehensive face-to-face presentation of the main content, purpose, meaning, and rules of engagement for the intervention, the participants will receive education through intervention tiers based on the TTM and the stages of the behavioral change model.

#### Control group

The participants assigned to the control group will receive TAU based on recommendations provided by their healthcare provider, including medication adherence, scheduled follow-up appointments, and lifestyle guidance. Moreover, to ensure objectivity in the study, the control group participants will remain unaware of the existence of the intervention group throughout the entire duration. To prevent bias, forms and information provided to the control group participants will not mention the TTM intervention. This precautionary measure intends to examine the additional benefits of the TTM intervention compared with the standard TAU.

*Tier 1 of the intervention*: *Study nurses’ assessment of the participants’ stages of behavioral change*. TTM will be employed in conjunction with an initial assessment of the participants’ stages of behavioral change. This assessment will analyze the participants’ willingness and need to modify their behavior and subsequently provide targeted behavior support intervention strategies tailored to their stages of behavioral change (see Table 1).

**Table 1:** Assessment of the participants’ stages of behavioral.

changeAssessment Content	Stages of Behavioral Change	Phase Status
Can you keep up with your weekly studies?	Pre-contemplation: The participant has no intention of changing the undesirable behavior within the next six months.	The participant may not be aware that changes need to be made or refuse to make any changes at this time.
Can you keep in touch with your healthcare provider?	Contemplation: The participant is ready to change the undesirable behavior and adopt a healthy behavior within six months.	The participant is aware of the need to change and is considering the benefits and drawbacks of making the change.
Can you take your medication regularly?	Preparation: The participant plans to adopt the behavior in the next 30 days and abandon the undesirable behavior.	The participant has made a commitment to change and is actively preparing for the behavioral change.
Can you maintain a good lifestyle?	Action: The participant has established and maintained healthy behaviors for six months.	The participant is actively involved in achieving and maintaining the desired behavioral change.

*Tier 2 of the intervention*: *Content of the behavioral change plan*. To elucidate the application of the TTM in bolstering self-management interventions, it is imperative to more explicitly integrate the principles of the model into the intervention’s multifaceted components. The TTM serves as a scaffold to comprehend the sequential stages an individual traverses in modifying behavior. Effective application necessitates bespoke interventions that cater to the participant’s extant stage of change, providing nuanced support that acknowledges their preparedness to embrace new behaviors. An elaborated schema for application includes the following (see Table 2).

**Table 2:** The behavioral change plan.

Phase of Change	Intervention Theme	Measures	Detailed Measures
Precontemplation	1. Awareness Arousal	Understand self-management behaviors and new perspectives	Personal health records for epilepsy and good cooperative relationships with the participants will be established, and their current disease treatment status will be clarified.
			1.2 The therapeutic principles of the intervention will be presented, with clear treatment goals and points of cooperation.
			1.3 The participants will be briefed on the importance of epilepsy self-management and its significant impact on quality of life in a face-to-face format, allowing the participants to understand the benefits of self-management behaviors, build confidence in disease treatment, and transform consciousness and behavior.
			1.4 Disseminate pedagogical resources and monitor engagement with educational content.
	2.Vivid Relief	Identify wrong thinking and change misrepresentation	
			2.1 The participants will recall how they felt during the onset of the disease through pictures and videos.
			2.2 The participants will share what they know about epilepsy and why they cannot manage themselves.
			2.3 The relevant reasons will be analyzed to find the motive for behavioral change.
Contemplation	3.Consciousness Aroused Again	Strengthen positive thinking and improve cognitive level	3.1 The successful cases of past participants’ self-management will be compared with those of failure to arouse the participants’ emotions about treating the disease.
			3.2 Awareness of the participants’ emotional arousal will be elucidated to change their behavior so that the correct beliefs of the participants are evaluated positively and the importance of self-management and its impact on quality of life is realized.
	4.Environmental Evaluation	Evaluate the participants’ behavior and correctly understand their behavior	4.1 The participants will evaluate their current self-regulatory behavior and recognize the importance of behavioral change.
			4.2 The participants will compare their physical conditions, good living condition, and poor times to guide them in making the right decisions. The participants will recognize their cognitive and behavioral inconsistencies and point to behaviors that are detrimental to disease control.
Preparation	5.Environmental Evaluation	Evaluate the external impact factors	5.1 The research nurses will strengthen humanistic care and focus on and understand the mental state of the participants.
			5.2 Participants will engage in introspection regarding the present environmental and social determinants that hinder effective disease management. Under the expert guidance of healthcare professionals, they will endeavor to amend these circumstances where feasible. Additionally, they will initiate dialogue with family members to facilitate positive behavioral modifications.
	6.Self-liberation	Form new beliefs and guide new behaviors	6.1 The participants will be motivated to commit themselves to epileptic self-management behaviors. The participants will state in detail the specific manifestations of the seizures and the factors that induce them. The participants will be asked to select at least one aspect that they consider to be poorly done, then discuss the contributing and impeding factors and propose goals and strategies for behavioral change.
			6.2 Participants will be apprised of their individual objectives by the research nurse and will subsequently formulate a scientifically grounded behavioral modification plan. This plan will encompass meticulous seizure monitoring, stringent adherence to medication regimens, and the adoption of lifestyle alterations, all of which are to be implemented expeditiously.
			6.3 The study nurses will help the participants identify preconceived symptoms of seizures and trigger factors and help the participants to actively adopt personal strategies (e.g., behavioral, cognitive, and emotional therapy).
	7.Social Liberation	Present the correct view of the disease	7.1 To make the participants aware that chronic disease management is a viable and acceptable concept in today’s society, a self-made Participants’ Guide to the Self-Management of Epilepsy will be issued to the participants.
			7.2 Evaluate proficiency in self-management competencies and participate in constructive communication with research evaluators. This involves maintaining consistent telephone correspondence with study evaluators, soliciting information as required, liaising with neurologists, and supervising both the formulation and execution of action strategies.
Action	8.Helping Relationships	Reduce stigma and build confidence in treatment	8.1 Successful experiences of people with epilepsy will be shared and social support will be sought to help the participants change and consolidate their behaviors.
			8.2 The participants will attend the online education course on epileptic self-management behaviors.
	9.Counter-conditioning	Consolidate coping with patterns and reinforce behavioral change	9.1 The participants will be assisted in setting short-term goals, long-term goals, interest schedules, exercise plans, diet records, etc. to replace unhealthy behaviors with beneficial behaviors.
			9.2 Conduct routine evaluations of data pertaining to self-management activities, seizure incidents, medication compliance, quality of life metrics, and adherence to program protocols.
	10.Intensifying Management		10.1 The family will give the participants material or moral rewards for achieving correct self-management behaviors.
	11. Stimulus Control		11.1 The participants will reduce bad behaviors, provide daily psychological cues, and put planning in a prominent position.
Maintenance Phase	12. Intensify Management	Consolidate coping patterns and reinforce behavioral change	12.1 The incidence and life status of the participants before and after their self-managing behaviors will be compared so that the participants will be motivated to adhere to their self-managing behaviors.
			12.2 The participants will be encouraged to persist in self-management for a long time, and discuss with family members how to participate with and encourage and reward the participants.
	13. Avoid a Return to Previous Behaviors	Avoid the recurrence of the participants’ perceptions and behaviors	Offer sustained support to maintain behavioral modification, which may include comprehensive training programs and continuous educational opportunities.
			13.1 The participants will be instructed to follow up regularly and return on time with a positive mindset to encourage the participants to adhere to treatment.
			13.2 The subsidence of self-management will be prevented through telephone follow-ups and family and participant supervision.
			13.3 In the event of a return to previous behaviors, the cause will be found and resolved in a timely manner.

1. Precontemplation: At this stage, participants are often unaware of the necessity for change. Motivational interviewing techniques will be employed to impart information that underscores the merits of self-management and incites a reevaluation of existing management behaviors, without pressuring immediate change.
2. Contemplation: Participants start acknowledging the potential for change but may experience ambivalence. Here, the intervention will supply tailored information concerning how self-management can enhance their quality of life. Facilitating a cost-benefit analysis of current behaviors versus the potential benefits of change, utilizing standardized scales, can assist in evaluating readiness for change.
3. Concomitant with these stages, data is systematically gathered and scrutinized to monitor adherence and efficacy. Regular interdisciplinary team meetings are vital to review individual progress and iteratively refine interventions, ensuring alignment with the participant’s current phase in the TTM for optimal behavioral change facilitation.
4. Preparation: Participants express intent to initiate behavioral change in the near term, typically within one month. The provision of tools and guidance will enable them to set realistic goals and devise a detailed action plan, acknowledging and strategizing around potential obstacles.
5. Action: At this stage, participants are actively involved in adopting self-management behaviors. The intervention reinforces these behaviors–such as maintaining seizure diaries, adhering to medication schedules, and applying stress management techniques–by offering actionable resources and consistent follow-up for feedback and positive reinforcement.
6. Maintenance: Participants have sustained their behavioral changes over a substantial time frame, aiming to prevent relapse. The intervention perpetuates support for these behaviors through ongoing intensive sessions, continuing education programs, and support networks.

*Tier 3 of the intervention*: *Behavioral change program health education lecture*. The Behavioral Change Program Health Education Lecture will be delivered weekly through videos, (see Table 3). To ensure comprehensive viewing of the health education video by participants, the following measures will be implemented: (1)Prior to commencing the intervention, participants’ current stage in the Transtheoretical Model (TTM) will be assessed through a brief conversation. This assessment will determine their level of interest and willingness to engage with the educational content. (2)The importance and relevance of the videos to participants’ health will be emphasized. Emphasis will be placed on how the information provided in the videos can assist them in making informed decisions about their health, specifically preventing seizures or managing epilepsy. (3)A specific date and time for weekly video viewing sessions will be scheduled. Reminders will be sent one or two days prior to the scheduled viewing time, aiming to integrate video viewing into participants’ daily routines and reduce the likelihood of sessions being forgotten or ignored. (4)The videos will be uploaded through "WeChat," a widely used electronic communication software among the Chinese population. This platform ensures convenient access for individuals to watch the videos repeatedly, particularly beneficial for those with time constraints or limited comprehension abilities. Recognizing that some participants may face barriers to video viewing due to mild or moderate cognitive impairment, we will encourage their family members or partners to watch the videos together. This collaborative approach will enable an assessment of both participants’ and their families’ understanding of the content. Additionally, comprehension will be checked after each viewing by addressing any questions or concerns raised, and positive reinforcement will be provided to acknowledge their engagement.

**Table 3:** The behavioral change program health education lecture.

Course Modules	Curriculum Arrangements	Course Content
Pilot Courses	Lesson 1	1.1 Reducing Disease Stigma: Specific examples of individuals with epilepsy who have achieved notable success in various fields or have become advocates for epilepsy awareness will be given. This will help inspire the participants and provide role models to reduce stigma.
		1.2 Improving Mental Resilience: The participants will share their personal experiences of and emotions about epilepsy, discuss their anxiety, and determine coping strategies.
		1.3 Sharing the Right Channels: Accurate popular science websites and public numbers will be given to the participants.
Disease Knowledge	Lesson 2	2.1 Eliminating Misconceptions about Epilepsy: Epilepsy will be introduced through fairy tales to explain the common misconceptions about epilepsy, such as thinking that all seizures are the same or that epilepsy is infectious. Clear and accurate information will be provided to help the participants understand the actual situation.
		2.2 Familiarity with Epilepsy Basics: The causes of seizures and the different types and frequency of seizures will be included to make the participants “specialists” in epilepsy and increase their confidence in the treatment of the disease.
		2.3 Getting the Right Recording Method: The participants will be taught how to properly record pre-seizure preconceptions, inducing factors, seizure performance, number of seizures, and specific time of seizures.
Diagnosis and Treatment Knowledge	Lesson 3	3.1 Familiarity with Epilepsy Diagnosis and Treatment: Tests that diagnose epilepsy to help diagnose/improve seizures, reduce concerns about medical procedures, and actively cooperate with treatment will be included.
		3.2 Handling Emotional Responses to Epilepsy: The emotional impact of receiving an epilepsy diagnosis will be addressed, and the participants will be provided with information on support resources (such as support groups and counseling services) so that they can discuss anxiety and stress management strategies related to the diagnosis.
		3.3 Familiarity with Epilepsy Treatment: An overview of anti-epileptic drugs and other adjuvant treatments will be given.
		3.4 Mastery of Drug Management Methods: The participants will be taught how to record the side effects of drugs, take anti-epileptic drugs with them when they are away, inability to change the use of drugs on their own, require the consent of a doctor when taking other medications, get to the hospital in time after a significant increase in the frequency of attacks, etc.
Cognitive Behavioral Therapy	Lesson 4	4.1 Mastery of Cognitive Behavioral Therapy: The participants will be informed of the common inducing factors and deflect their attention through cognitive behavioral therapy (e.g., self-alluding, self-relaxing, deep breathing, etc.) to avoid seizures as much as possible.
		4.2 Mastery of the Principles of First Aid for Epilepsy: Through behavioral therapy, the participants and their families will master the principles of first aid before seizures occur. The intervener will watch videos with the participants, combine the videos for role-playing and behavioral drills, add some simulated real-world social situations, and allow the participants to discuss and practice using these skills in real life.
Epilepsy and Life	Lesson 5	5.1 Personal Life after Illness: Precautions for personal life (e.g., using an electric knife alone) will be introduced to share how epilepsy affects personal and professional relationships, post-epilepsy rules on hobbies, exercise, driving, and employment, and how to seek psychological support for anxiety.
		5.2 Social Life after Illness: Tips for playing outside, how to explain epilepsy to others, and what happens after good disease control will be introduced, reducing the need to restrict social interaction to build and maintain social contact, reducing the participants’ feelings of isolation and stigma, and pursuing confident communication.
Epilepsy Management for Women	Lessons 6	6.1 Fertility and Family Planning: Provide consultations on family planning and reproductive health that encompass fertility counseling, contraceptive options, epilepsy management during pregnancy, and lactation guidance.
		6.2 Specialized counselling and support: Counselling services for women with epilepsy to cope with gender-related psychosocial stresses, (e.g. family responsibilities and work-life balance pressures).
		6.3Targeted treatment planning and education: Physicians should take into account the effects of epilepsy medications on women at specific times (e.g., medication selection and dosage adjustments during pregnancy, effects of the menstrual cycle on epilepsy, etc.) and provide counseling and guidance accordingly.
		6.4 Promoting healthy lifestyles: Encouraging healthy lifestyles, including moderate physical exercise, a balanced diet and adequate rest, are particularly beneficial to women’s health management.
Intensive Courses	Lessons 7–8	To prevent the participants from returning to the previous stage and forgetting their behavioral change plan, the participants will be given monthly knowledge enhancement and consolidation, including epilepsy-inducing factors, identification of precursors, first aid principles, regular medication, and on-time referral.

### Outcomes

#### Baseline

At baseline (T_0_), the participants’ socio-demographic data will be collected, including gender, age, ethnicity, marital status, education level, occupation, and economic level, through specially designed questionnaires. In addition, a web-based survey will be utilized to gather data on the participants’ previous self-management behavior with the Chinese version of the Adult Epilepsy Self-Management Scale (C-ESMS), quality of life with the Quality of Life in Epilepsy Inventory-31 (QOLIE-31), and psychological status with the Hospital Anxiety and Depression Scale (HADS). It is important to highlight that the independent evaluation researchers will be responsible for securely storing all collected data to ensure consistency and standardization throughout the data collection process.

#### Primary outcome

The primary outcome indicator for both groups will be the change in self-reported self-management ability over the previous month between baseline and six-month follow-up using the C-ESMS [31] and the Liverpool Seizure Severity Scale (LSSS 2.0) [32]. The C-ESMS is a validated 38-item self-report scale commonly used for individuals with epilepsy. It consists of five subdomains, namely, medication management (e.g., good medication compliance; 10 items), information management (e.g., keeping comprehensive seizure records; eight items), safety management (e.g., avoidance of alcohol; eight items), seizure management (e.g., timely communication with the doctor during increased seizure frequency; six items), and lifestyle management (e.g., stress management; six items). Additionally, the LSSS 2.0 is a self-reported questionnaire designed to assess epilepsy severity and detect changes in seizure severity over time, and the Chinese version of the LSSS 2.0 will be utilized in this study.

#### Secondary outcomes

The participants will be required to complete three assessments, namely, the QOLIE-31 and the HADS., to measure medication adherence. The QOLIE-31 is a self-reported inventory that evaluates a patient’s quality of life, epilepsy-related concerns, and overall well-being over the previous four weeks, and it is comprised of 31 items that assess various dimensions, such as seizure worry and emotional well-being. This study will employ the Chinese version of the QOLIE-31, which has demonstrated good generalizability and has been extensively utilized to assess the living conditions of Chinese individuals with epilepsy. The HADS is a widely employed self-report scale that consists of two subscales: HAD-A for measuring anxiety and HAD-D for measuring depression. This scale has achieved satisfactory internal reliability and construct validity and has been widely adopted in both clinical and non-clinical settings in China.

### Embedded qualitative interviews

Following the implementation of the intervention, qualitative interviews will be conducted to elucidate participants’ perceptions and lived experiences related to the intervention. A purposive sampling strategy will be employed to select interviewees from among the 80 members of the intervention cohort. These individuals will be preliminarily engaged via a succinct telephone communication to schedule the interviews, which will be tailored to address the research objectives. The average duration of each in-depth interview will be ascertained and communicated to participants during scheduling to set clear expectations and ensure adequate time allocation for comprehensive discussions. Eligibility for participation will hinge upon two primary criteria: (a) the successful completion of all prescribed phases of the intervention, and (b) the expressed consent to partake in the post-intervention interviews. To capture a heterogeneous array of perspectives, we will endeavor to ensure an equitable distribution of participants across various demographic profiles. Interviews will be executed by a duo of adept research evaluators, trained in qualitative methodologies. These evaluators will conduct one-on-one, semi-structured dialogues, drawing upon a pre-established compendium of inquiry prompts as delineated in Table 4. Prior to the commencement of each interview, explicit consent will be solicited from participants for the audio recording of the exchange to guarantee the veracity of the data.

**Table 4:** Interview questions for the qualitative evaluation of the intervention participants’ behavioral change plans.

1. What new insights, if any, have you gained about your health after participating in the intervention?
2. Can you describe how the intervention has affected your management of epilepsy symptoms in your daily life?
3. What do you think of the length of the intervention in terms of affecting your behavioral changes?
4. What challenges, if any, did you encounter during the intervention and how did you cope with them?
5. In what ways, if any, do you feel the intervention affected your quality of life and well-being?

Subsequent to the interview, within a 24-hour window, audio recordings will be meticulously transcribed verbatim. To verify the fidelity of the transcription, the resultant textual renditions will be reviewed by the respective interviewees for confirmation. Data collection via interviews will continue until the point of data saturation, where no additional novel insights are discerned. For the coding and analytical phases, we will adopt an iterative process that encompasses data reduction, data display, and corroboration of results through thematic categorization. NVivo 11, a sophisticated qualitative data analysis software, will be deployed to facilitate the data reduction process, allowing for an efficient and comprehensive analysis.

### Data collection, management, and analysis

#### Timeline

The researchers will begin the research at baseline, and then continue the study at one-month, three-month, and six-month intervals (the margin will be ± 1 week) by collecting electronic questionnaires (the participants will no longer have to book an outpatient appointment) to minimize the participants’ burden. The electronic questionnaires will be disseminated using a professional online tool that will operate on a secure server with regular system backups. The tool will store only encoded, non-personalized data in a separate and secure location, ensuring restricted access to others. In the event that a participant has difficulty comprehending a question or responding, the evaluator, who will be a trained staff member, will provide clarifications without indicating any potential answers or biases.

Table 5 provides an overview of the evaluation areas and corresponding timeline. The data collection process will utilize well-established tools that have previously been employed in clinical trials involving epilepsy patients. These tools will be chosen to ensure comparability across the studies and, if available, will utilize verified Chinese language versions to enhance their suitability for the target population.

**Table 5:** Evaluation timetable.

Instrument	Evaluation Area	Measures
General Information	Socio-demographics	Baseline
C-ESMS [31]	Changes in drugs, information, safety, seizures	Baseline and follow-up sessions *
HADS [33]	Severity of anxiety and depression	Baseline and follow-up sessions *
LSSS [34]	Severity of seizures	Baseline and follow-up sessions *
QOLIE-31 [35]	Health-related quality of life	Baseline and follow-up sessions *

*Follow-up sessions: post-intervention (one to seven days after the last intervention) and three-month and six-month follow-up (three and six months after the last intervention (±2 weeks)); C-ESMS = Chinese version of the Epilepsy Self-Management Scale; HADS = Hospital Anxiety and Depression Scale; LSSS = Liverpool Seizure Severity Scale; and QOLIE-31 = Quality of Life Scale in Epilepsy-31.

### Intervention allocation: Sequence generation, distribution of hidden mechanisms, and implementation

To uphold the integrity and blinding of the study, a 1:1 allocation to control or intervention groups was carried out using a computer-generated randomization sequence. This sequence was created using SPSS version 29.0, ensuring an equal chance of assignment to either group with no additional restrictions such as blocking. Stratification was based on the annual frequency of epileptic seizures, creating two strata of ≥12 times or <12 times per year [36], to guarantee a balanced distribution across groups before randomization.

The random allocation sequence was implemented through sequentially numbered, opaque, sealed envelopes (SNOSE) and managed by an independent research team, who were not involved in participant enrollment or intervention administration. This method, alongside a secure electronic system, was employed to preserve the sequence’s concealment. Participants were enrolled by a designated team of researchers, distinct from the independent team, who further assigned participants to interventions according to the concealed allocation sequence.

To ensure blinding, interventions were designed to be indistinguishable in terms of appearance and delivery to participants across both control and intervention groups. This measure, coupled with the secure management of the allocation sequence, guaranteed that all participants and outcome assessors were blind to group assignments, thus preventing selection bias and assessment bias. Knowledge of group assignments was withheld from participants and outcome assessors until after the statistical analysis of the primary outcomes was complete, at which point the coded group assignments were disclosed. Although care providers were aware of the treatment allocation, their influence on the study’s outcomes was mitigated by the blinding of participants and independent assessment of outcomes, alongside employing a rigorous strategy for randomization. Independent researchers managed the allocation sequence, helping to minimize bias and maintain the study’s integrity during both the allocation process and the data analysis phase, ensuring the validity of the study results.

### Sample size

Effect sizes (Cohen’s d) in self-management studies typically fall below 0.8. Previous research has identified effect sizes of d = 0.79 [37] or d = 0.7 [38]. However, due to variations in the intervention strategies and outcome measures of these studies, we will conservatively assume a medium effect size of d = 0.5 to achieve 80% statistical power with a two-tailed significance level of 0.05 [39]. Consequently, it is recommended to recruit a minimum of 128 participants. Anticipating a 20% attrition rate in follow-up participation, this study aims to enroll approximately 160 participants. However, significant measures will be implemented to maintain a participant retention rate of less than 5%, as follows: (1) minimizing burden and inconvenience through minimal data collection and utilizing network-based methods; (2) acquiring accurate contact information of the participants to prevent loss to follow-up; (3) implementing real-time monitoring for data collection; (4) employing educational strategies, such as reminders and dedicated websites, to enhance participant participation and engagement; and (5) providing treatment incentives in the form of financial compensation ($5 per follow-up assessment). In addition, interim analyses will be conducted during the course of the study, where applicable, to monitor preliminary effectiveness and safety data. If the interim analyses show that the predetermined attrition rate is exceeded, we will reassess the retention strategy and adjust it accordingly. Discontinuation guidelines will include the identification of significant adverse events or evidence of study futility (e.g., conditional efficacy analysis of less than 20%) at the time of the interim analysis. These measures are intended to ensure participant safety and uphold the integrity of the study.

### Statistical analysis

Initial statistical scrutiny will begin with an exploration of the ordinal data using descriptive statistics to determine its suitability for parametric or non-parametric methods in null hypothesis testing. Preliminary examination will include computing central tendencies—median and mode—and measures of dispersion, such as specifically the range and interquartile range. To support the potential use of non-parametric techniques, we will assess the data’s distribution for normality using either the Kolmogorov-Smirnov or the Shapiro-Wilk test.

We will closely examine outliers to discern whether they represent true variability or data entry errors, informing decisions on their inclusion or exclusion in the analysis. A thorough investigation of missing data will identify any patterns, ensuring gaps are managed to maintain the integrity of our study’s findings and tailor our approach to the type of data absence.

Given our data’s unique characteristics, we will remain flexible with our statistical methods. Any necessary revisions to our approach will be made as analysis proceeds. The statistical methods and their rationales will be detailed in our results paper, aligning our findings’ credibility with the data’s complexities.

Descriptive and inferential statistics will be conducted. The Wilcoxon signed-rank test will compare data within groups, while the Mann-Whitney U test will be used for between-group comparisons. The Friedman test will be utilized for repeated measures within groups to analyze ordinal data over time. We acknowledge that the Friedman test is not a direct equivalent to repeated measures ANOVA for continuous data but is appropriate for our non-parametric dataset. To gain insights comparable to those from repeated measures ANOVA, we will examine score changes using the Friedman or Kruskal-Wallis test, with post-hoc analyses using the Wilcoxon signed-rank or Mann-Whitney U test to handle multiple comparisons.

For subgroup analyses, we will use ordinal logistic regression with necessary adjustments to baseline variables, maintaining a two-tailed significance threshold of p < 0.05 for all tests. Insights from initial data assessments may prompt refinements in our analytical tactics.

All statistical procedures will be executed in SPSS, version 29.0. We will ensure meticulous documentation to promote transparency in our methodology and reproducibility of our study’s outcomes.

### Ethics and dissemination

This study will be conducted according to the Declaration of Helsinki and was approved by the Ethics Council of Zunyi Medical University (KLLY-2022-062). The trial results will be submitted to a peer-reviewed open-access journal for publication and disseminated through presentations at conferences.

## Discussion

The global incidence of epilepsy is escalating, and its distinct clinical manifestations frequently result in significant adverse outcomes, such as educational disruption, unemployment, and reproductive challenges, which profoundly impact affected individuals’ lives [5]. To combat these issues, our protocol introduces a randomized controlled design enriched with several novel and informative components.

Firstly, our study seeks to evaluate the effectiveness of self-management behaviors, anchored by the Trans-Theoretical Model (TTM), which offers a staged approach to behavior change. Providing a structured pathway following TTM guidance could promote meaningful behavioral modifications.

Secondly, by incorporating insights from previous randomized controlled trials, our protocol aims to establish methodologies that are both detailed and encompassing, enhancing the study’s validity and ensuring precise reporting of results.

Thirdly, we plan to implement multifaceted measures to appraise both the objective and subjective dimensions of key outcomes during the intervention and at extended follow-up intervals.

Lastly, qualitative interviews will be conducted to capture the intervention group participants’ perspectives, offering rich insights into their experiential narratives.

Despite these meticulous preparations, it is vital to acknowledge the study’s limitations. Conducted in a single tertiary hospital in Guizhou Province, Southwest China, our findings might not be entirely representative of the broader Chinese epilepsy population due to regional prevalence disparities. For broader applicability, future research should encompass diverse regions and include multicenter studies to enhance external validity.

The eligibility requirement of a minimum elementary education level may inadvertently exclude individuals with lesser educational attainments. Subsequent studies should aim to design educational content and interventions that are accessible to individuals across all educational backgrounds, thereby enhancing the study’s inclusivity and applicability.

Although the six-month follow-up period in our study offers insights into short-term effects, the durability of these effects remains uncertain. Future research should prolong the follow-up duration to assess the long-term efficacy and sustainability of the interventions, as well as to monitor any late-onset adverse events.

Resource constraints at the hospital have precluded the inclusion of objective biomarkers like serum drug levels. An over-reliance on subjective assessments may introduce a degree of bias. Future studies should seek to include a broader spectrum of objective biomarkers and clinical indicators to increase result reliability and provide a more holistic disease management assessment.

In light of these considerations and by addressing the aforementioned limitations, this study is poised to contribute valuable knowledge to the epilepsy management domain, aiming to ameliorate the lives of those afflicted by this condition.

Our goal is to ascertain the efficacy of a behavior-change intervention in fostering epilepsy self-management and seizure control among participants. Should the intervention prove efficacious, it would be a significant asset for adults with epilepsy. The adoption of this intervention by healthcare providers could ensure its sustained integration within the healthcare system, thereby improving the well-being and quality of life of those with epilepsy.

## Supporting information

S1 Checklist Checklist SPIRIT-checklist. (PDF)

S1 File The ethics committee (Chinese version). (PDF)

S2 File The ethics committee (English version). (PDF)
