# Supplementary material for: Effects of the transtheoretical model-based self-management program on behavioral change in persons with epilepsy: Study protocol for a randomized controlled trial
Source: PLoS One. 2024 Nov 25;19(11):e0305547. doi: 10.1371/journal.pone.0305547 (PMC11588227; doi:10.1371/journal.pone.0305547)
Supplement: S1 Checklist — (PDF) [file pone.0305547.s001.pdf]

SPIRIT

STANDARD PROTOCOL ITEMS: RECOMMENDATIONS FOR INTERVENTIONAL TRIALS

SPIRIT 2013 Checklist: Recommended items to address in a clinical trial protocol and related documents*

Section/item	Item No	Description
**Administrative information**		
Title	1	The effects of self-management interventions on behavioral change and seizure frequency in epileptic patients: Study protocol for a randomized controlled trial. (P1)
Trial registration	2a	ChiCTR2300074975
	2b	N/A,There is currently no data available
Protocol version	3	Spirit 2013 Guidance Last update: February 19, 2023(P7 line12)
Funding	4	(P27 line7-14)
Roles and responsibilities	5a	(P1 line11-20，P28 line1-7)
	5b	Cai Li, 1135387169@qq.com
	5c	HH, as a sponsor, has the ultimate right to publish the report(P28 line4-5)
	5d	n/a; the research group assumes the responsibility for overseeing the trial
**Introduction**		
Background and rationale	6a	(P3-6)
	6b	(P7-8)
Objectives	7	(P7 line5-8)
Trial design	8	(P7 line3-4)
**Methods: Participants, interventions, and outcomes**		
Study setting	9	(P7 line4-6)
Eligibility criteria	10	(P9 line1-15)

Interventions	11a	(P9-P18)
	11b	n/a; there are no anticipated harmful consequences of participating in the study
	11c	(P24 line3-9)
	11d	(P9 line17-20，P10-P11)
Outcomes	12	(P18 line26-27，P19，P20 line1-2)
Participant timeline	13	(P22 Table 5)
Sample size	14	(P23 line19-22，P24 line1)
Recruitment	15	(P23 line2-10)
**Methods: Assignment of interventions (for controlled trials)**		
Allocation:		
Sequence generation	16a	(P23 line7-10)
Allocation concealment mechanism	16b	(P23 line10-13)
Implementation	16c	Generate the allocation sequence(P23 line9-10)<br>Enrol participants(P8 line3-7)<br>Assign participants to interventions(P23 line13-15)
Blinding (masking)	17a	(P7 line3-8)<br>23
	17b	n/a; This study will not cause participants to experience emergency situations (such as serious adverse events)
**Methods: Data collection, management, and analysis**		
Data collection methods	18a	(P9-P10，P18-P20)
	18b	n/a; participation is voluntary and students are free to discontinue at any point in time
Data management	19	(P19 line23-25，P22-P23)
Statistical methods	20a	(P24 line11-20)
	20b	(P24 line11-20)

	20c	n/a; currently not considered
**Methods: Monitoring**		
Data monitoring	21a	n/a; no harmful consequences of participation are anticipated
	21b	n/a; no harmful consequences of participation are anticipated
Harms	22	n/a; no harmful consequences of participation are anticipated
Auditing	23	n/a; no harmful consequences of participation are anticipated
**Ethics and dissemination**		
Research ethics approval	24	(P25 line1-5)
Protocol amendments	25	n/a;If there are any changes, they will be communicated and modified to the experimental registration center and the journal
Consent or assent	26a	(P8 line3-10)
	26b	n/a; no ancillary studies are planned
Confidentiality	27	(P21 line18-20，P22 line1-8)
Declaration of interests	28	Additional information
Access to data	29	Additional information
Ancillary and post-trial care	30	n/a; no harmful consequences of participation are anticipated
Dissemination policy	31a	(P24 line3-5)
	31b	(P28)
	31c	N/a, confidentiality involved, can be transmitted through email
**Appendices**		
Informed consent materials	32	n/a; consent form can be provided by the corresponding author upon request
Biological specimens	33	n/a; no biological specimens are collected

*It is strongly recommended that this checklist be read in conjunction with the SPIRIT 2013 Explanation & Elaboration for important clarification on the items. Amendments to the protocol should be tracked and dated. The SPIRIT checklist is copyrighted by the SPIRIT

Group under the Creative Commons "Attribution-NonCommercial-NoDerivs 3.0 Unported" license.
