# Supplementary material for: Effects of the transtheoretical model-based self-management program on behavioral change in persons with epilepsy: Study protocol for a randomized controlled trial
Source: PLoS One. 2024 Nov 25;19(11):e0305547. doi: 10.1371/journal.pone.0305547 (PMC11588227; doi:10.1371/journal.pone.0305547)
Supplement: S1 File — (PDF) [file pone.0305547.s002.pdf]

遵义医科大学附属医院生物医学研究伦理委员会

伦理审查批件

伦理审查批件号：KLLY-2022-062

受理号	KLLY-2022-062		
项目名称	基于 TTM 理论对成年癫痫患者自我管理的影响研究		
项目来源	硕士学位论文开题项目		
项目负责人	李彩	硕士研究生导师	黄浩
项目负责人所在科室	护理学院		
审查类别	初始审查	审查方式	快速审查
审查时间	2022.12.31	审查地点	伦理委员会办公室
审查文件	1. 伦理审查申请书<br>2. 开题报告（版本日期：2022-06-30）<br>3. 知情同意书（版本号：V1.0；版本日期：2022-06-20）		

审查意见：

依据世界医学会《赫尔辛基宣言》、国际医学科学组织委员会《涉及人的生物医学研究国际伦理准则》、《药物临床试验质量管理规范》、《涉及人体的生物医学研究伦理审查办法》、《药物临床试验伦理审查工作指导原则》等法律、法规、规章、规范性文件和国际准则，经本伦理委员会审查，同意该项目按照预定方案开展研究。

请遵循伦理委员会批准的方案开展研究，保护受试者的健康和权益。

1. 研究过程中若变更项目负责人，对研究方案、知情同意书、病例报告表、调查问卷、招募材料等的任何修改，请提交修正案审查申请；

2. 请按照相关法律法规规定以及研究方案中对于安全性事件报告计划，及时向遵义医科大学附属医院生物医学研究伦理委员会提交书面不良事件报告；

3. 研究者没有遵从方案开展研究，可能对受试者的权益/健康以及研究的科学性造成不良影响，请提交违规事件报告；

4. 申请人暂停或提前终止临床研究，请及时提交暂停/中止研究报告；

5. 研究结束时，请提交结题报告。

6. 请至少在失效日期前 1 个月提交持续审查申请。

批准日期	2022.12.31	批件失效日期	2024.12.30

伦理委员会

科研项目伦理审查申请书

项目名称：基于TTM理论对成年癫痫患者自我管理的影响研究

项目负责人：李彩

科研项目类别：

国家级☐　省部级☐　市级☐　局级/校级☑　其他☐

项目所在本院科室（专业）：神经内科（护理）

填表日期：2022 年 12 月 27 日

项目名称	基于TTM理论对成年癫痫患者自我管理的影响研究		
项目来源			
开题报告版本号	V1.0	开题报告<br>版本日期	2020.9.27
知情同意书<br>版本号	V1.0	知情同意书<br>版本日期	2020.9.27
申办方或项目发起单位			
联系人	李彩	联系电话	
组长单位			
组长单位主要研究者	李彩、黄浩		
参加单位			
本院承担科室	神经内科		
本院主要研究者	李彩、黄浩		

一、研究信息

- 科研目的：

①探讨基于TTM理论制定成年EP患者自我管理的方案比传统策略指导下的EP慢病管理是否具有优越性；

②探讨该方案对提升EP患者生活质量，改善不良心理状况，提高疾病自我管理能力的作用。

方案设计类型

- ☑实验性研究

☐ 观察性研究：☐ 回顾性分析，☐ 前瞻性研究

☐ 利用人体生物标本的研究：☐ 以往采集保存，☐ 研究采集

- 可能出现的不良反应及防治措施（请说明）：
- 要求具备的特殊条件

☐重症监护 ， ☐隔离区 ，☐手术 ， ☐儿童重症监护 ， ☐静脉输注 ，

☐计算机断层扫描 ， ☐妇科 ， ☐基因治疗 ， ☐管制药品（麻醉药/精神药），☐其他（请具体说明）：

____

- 研究期限： 自2022年6月至 2024年6月
- 其他研究信息：
  - ✧ 资金来源：☐ 企业，☐ 政府，☐ 学术团体，☐ 本单位，☑ 自筹
  - ✧ 该研究项目是否被其他伦理委员会拒绝或否决过？

    ☑ 无，☐ 有→请提交相关文件
  - ✧ 该研究项目是否曾被其他伦理委员会暂停或者终止过？

    ☑ 无，☐ 有→请提交相关文件
  - ✧ 本院科室是否有同类科研项目在研？☐是，☑否
  - ✧ 本院科室目前在研项目中，与本项目的目标疾病相同的项目数：____项。
  - ✧ 研究需要使用人体生物标本：

    ☑ 否，

    ☐ 是→填写下列选项
    - ➤ 采集生物标本：☐ 是，☐ 否
    - ➤ 利用以往保存的生物标本：☐ 是，☐ 否
- 招募受试者：
  - ✧ 谁负责招募：☐ 医生，☐ 研究者，☐ 研究助理，☑ 研究护士，

    ☐ 其它：____
  - ✧ 招募方式：☐ 广告，☐ 诊疗过程，☑ 数据库，☐ 中介，☐ 其他：_______
  - ✧ 招募人群特征：

☐健康者 ，☐患者（请描述）：____成年癫痫患者________

受试者年龄范围：_≥18岁____ 受试者性别：__无特殊要求_____________

受试者（☐是，☑否）含弱势群体：________（若有，请标注所涉及的弱势群体。如儿童/未成年人、认知障碍或健康状况而没有能力做出知情同意的成人、申办者/研究者的雇员或学生、教育/经济地位低下的人员、疾病终末期患者、囚犯或劳教人员、孕妇等）

  - ✧ 受试者报酬：☐ 有，☑ 无
    - ➤ 报酬金额：（可填写）___________
    - ➤ 报酬支付方式：☐ 按随访观察时点，分次支付，

      ☐ 按完成的随访观察工作量，一次性支付，

☐ 完成全部随访观察后支付

- 知情同意的过程：
  - ✧ 谁获取知情同意：☐ 医生/研究者，☐ 医生，
    ☑ 研究者，☐其它：________________
  - ✧ 获取知情同意地点：☐ 私密房间/受试者接待室，☐ 诊室，☑ 病房，
    ☐ 其它：____________________________
  - ✧ 知情同意签字：☑受试者签字，☐法定代理人签字，☐监护人签字
- 知情同意的例外：
  ☑ 否，
  ☐ 是→选择以下选项：
  - ✧ ☐申请开展在紧急情况下无法获得知情同意的研究：
    - ➢ 研究人群处于危及生命的紧急状况，需要在发病后很快进行干预；
    - ➢ 在该紧急情况下，大部分病人无法给予知情同意，且没有时间找到法定代理人；
  - ✧ ☐申请免除知情同意——利用以往临床诊疗中获得的病历/生物标本的研究；
  - ✧ ☐申请免除知情同意——研究病历/生物标本的二次利用；
  - ✧ ☐申请免除知情同意签字——签了字的知情同意书会对受试者的隐私构成不正当的威胁，联系受试者真实身份和研究的唯一记录是知情同意文件，并且主要风险就来自于受试者身份或个人隐私的泄露；
  - ✧ ☐申请免除知情同意签字——研究对受试者的风险不大于最小风险，并且如果脱离“研究”背景，相同情况下的行为或程序不要求签署书面知情同意，如访谈研究，邮件/电话调查

二、项目研究人员

1、本院：

主要研究者：______________职称：____________________

科室：______________联系电话：__________________

参加者：

（1）主要联系人：__________职称：________________

科室：______________联系电话：______________

（2）其他参加者：*（如涉及其他科室人员，请注明其科室）*

______________ 职称：______________

科室：______________ __________分工

______________ 职称：______________

科室：__________ __________分工

__________ 职称：__________

科室：__________ __________分工

__________ 职称：__________

科室：__________ __________

2、其它单位*（如涉及，请填写以下内容）*：

①____________________

主要研究者：__________ __________职称：

②____________________

主要研究者：__________ __________职称：

③____________________

主要研究者：__________ __________职称：

研究者责任声明	我将遵循研究方案以及伦理委员会的要求，开展本项临床研究		
主要研究者签字		日期	
科室主任签字		日期	

伦理应用试验方案

提要
**资金来源名称：** 本课题得到了国家自然科学基金项目（No.81760247；82171450）、贵州省教育厅青年科技人才培养项目（No.KY（2017）202）、遵义医学院神经病学研究生工作站（No.GZZ2017004）的资助，遵义医科大学附属医院博士生科研基金（批准号：（2016）14）和贵州省科技基金【2019）1350号】，
**研究名称：** 基于行为阶段转变理论对成年癫痫患者自我管理的影响研究
**计划研究地点：** 遵义医科大学附属医院
**参与者人数：** 160人
**主要负责人：李彩**
**研究时间：** 2023年8月 - 2024年 3月
**目的：**<br>①构建行为阶段转变理论指导下的成年癫痫患者自我管理干预方案<br>②验证行为阶段转变理论指导下的成年癫痫患者自我管理干预方案<br>③调查成年癫痫患者对自我管理方案的效果评价
**研究设计：本研究是一项随机、参与者盲法、对照的临床试验。**
**研究标准：**<br>纳入标准：<br>①符合2017年修订版国际抗癫联盟(ILAE)公布的癫痫诊断标准；<br>②病程≥6个月且期间至少有1次癫痫发作；<br>③服用至少一种抗癫痫药物；<br>④年龄≥18岁；<br>⑤小学以上文化程度；<br>⑥长期居住在本市；<br>⑦能接受干预方案，知情同意并自愿参加。<br>排除标准：<br>①有脑外科手术史；<br>②诊断为急性神经系统疾病或物质滥用相关的急性症状性癫痫发作；

③合并其他神经或精神疾病、智力及人格障碍，不能正常交流；<br>④有严重的心、肝、肾等躯体疾病，合并有恶性病变、进行性或变性疾病；<br>⑤正在参加其他临床实验者。<br>脱落标准:<br>①在研究期间转到其他医院治疗者；<br>②未完成全部干预和数据收集者；<br>③自愿退出研究者。
**样本量**: 此次研究采用 G-power3.1 软件计算样本量，选择主要统计分析方法为T test，并在软件中设N1/N2=1， α=0.05，effectsize=0. 5，Tail为双侧，Power=0.80，计算出总样本量为 128 例。由于存在失访可能增加了 20%，计算样本量为 160 例。

研究方案构建

1.癫痫患者自我管理干预方案的初步构建

系统回顾国内外相关文献及指南，筛选并提取国内外癫痫自我管理的相关指标及影响因素，将以往文献研究成果、相关研究理论与癫痫患者的疾病特征相结合，初步构建自我管理干预方案初稿。

(1) 系统性文献回顾癫痫自我管理的影响因素、评估工具及干预方案

我们将根据各数据库特点制定检索式，采用布尔逻辑词连接各检索词，通过检索原始研究提取与癫痫自我管理有关的内容，

检索原始研究纳入排除标准 ：

纳入标准：①研究对象为≥18岁的成年癫痫患者；

②研究内容涉及癫痫自我管理及其影响因素及干预措施；

③语言为中文或英文。

排除标准：①综述；

②无法获取全文；

③重复发表的文献。

(2) 筛选方法

由 2 名研究人员独立完成文献筛选过程，首先初步阅读文题和摘要，排除显著不相关的文献后，进一步阅读全文，剔除不符合纳入标准的文献，若信息不全，联系作者获取，若产生分歧，与团队负责人讨论决定。

利用Cochrane纳入的RCT文献质量评价(风险偏倚评估工具)中文版对原始研究进行证据等级评价，纳入偏移风险较低的原始研究。使用Excel 2016录入文献基本信息及癫痫自我管理干预内容、观察指标、影响因素。

(3) 纳入风险较低、结局良好的RCT构建干预方案

通过Excel 2016汇总相关数据，总结有关于癫痫自我管理的干预者、干预方法、干预效果的内容，提取干预结局良好的原始研究形成本次研究干预方案的初稿。

2.癫痫患者自我管理干预方案的修订

邀请癫痫领域的医护专家开展专家咨询，通过德尔菲法讨论初步构建的癫痫患者自我管理干预方案的科学性及可行性，结合专家给予的反馈意见再次进行修订与完善，最终形成干预方案。

（1）专家纳入标准

医疗专家纳入标准：①具有硕士研究生及以上学历；

②副高级及以上职称；

③在神经内科领域工作满 10 年及以上；

④自愿参与本研究专家咨询。

护理专家纳入标准：①具有本科及以上学历；

②副高级及以上职称；

③在神经内科领域工作满 5 年及以上；

④自愿参与本研究专家咨询。

（2）确定专家人数

根据专家工作领域的同质性，选择10名专家进行咨询。

（3）专家咨询表设计

包括 3 部分：

①致专家信（说明研究目的与意义、填表说明、期望完成的时间、对专家的致谢等）；

②专家基本情况调查（专家基本资料情况、对咨询内容的熟悉程度自评和判断依据等）；

③咨询主体内容（干预方案各条目内容栏、条目重要度评分栏、修改意见栏、新增条目栏等）。

（4）专家咨询的实施步骤

向专家简要说明研究目的、意义、任务等，在征得同意后使用问卷星发放咨询表进行第一轮专家咨询填写，待填写完毕后及时回收。将各位专家的意见加以整理，再进行第二次专家咨询，待意见趋于一致则停止咨询，形成最终版癫痫自我管理干预方案。

（5）统计分析

从问卷星平台导出数据，使用 SPSS 29.0 进行统计学分析：

①专家一般资料：计量资料采用均数、标准差表示，计数资料采用频数、百分比表示；

②专家积极性使用问卷回收率表示；

③专家权威程度使用权威系数（Cr）表示；

④专家意见的集中程度使用条目的均数和满分频率表示；

⑤专家意见协调程度使用变异系数、肯德尔和谐系数（Kendall’s W）表示。

3.基于行为阶段转变理论癫痫患者自我管理干预方案的实证研究

(1) 研究对象

于研究时间内随机选取就诊于遵义医科大学附属医院神经内科门诊部符合纳入标准的癫痫患者作为研究对象。干预前对所有患者的基线资料进行评估，包括一般资料、生活质量、自我管理情况等，根据随机数字表法分为试验组对照组，对其进行为期六个月的干预研究，对照组实施常规癫痫健康教育方法，试验组采用基于跨理论模型的健康教育模式进行自我管理行为干预，干预前、干预 1、3、6 个月，分别根据癫痫自我管理量表(ESMS)、癫痫患者生活质量量表（QOLIE-31）、癫痫发作次数来评估两组患者的行为改变阶段以及自我管理行为干预效果，进而对基于TTM理论成年癫痫患者自我管理干预方案进行效果评价。

(2) 研究方案

量性研究

干预前采用面对面问卷调查方式，对符合纳排标准的研究对象，由培训合格的调查员现场发放纸质问卷或电子问卷，被调查对象自行填写，因文化程度限制不便独立完成问卷者，由调查员读题，被调查员作出自评。所有问卷当场收回保证真实性、有效性。

本研究实行单盲法，即研究对象不了解具体的分组情况。本研究将研究对象按进入本研究的顺序进行编号，遵循 1:1 的比例，采用分层随机化的方法（每年癫痫发作≥12次或＜12次），研究人员在医生协助下使用随机数字表进行随机分组，使用信封法在研究开始前研究人员和患者对于将会分在哪一组不可预知，旨在保证患者进入各组的机会相等，尽可能使得两组匹配到的一般人口学特征，以便2个干预组之间平均分配癫痫发作的严重程度，减少偏倚可能性。

1） 对照组：常规癫痫健康教育

时间：进行为期6个月的常规治疗及健康咨询，门诊复诊1次/月，30 分钟/次。

内容：

①向患者讲解疾病的相关知识，分发《癫痫疾病宣传教育手册》，针对患者的问题进行解答；

②每月按时复诊，有问题及时就诊。

2） 试验组：基于跨理论模型（TTM）的自我管理健康教育

时间：进行为期6个月的干预，1次/月，共计 6 次，30-60 分钟/次；门诊复诊1次/月，30 分钟/次，电话随访1次/月，20 分钟/次，共计 6 次。

形式：基于TTM理论进行与对照组相同的健康教育干预，除对照组内容外增加TTM阶段评估后进行个性化健康教育及自我管理计划拟定，每月电话随访。

内容：

①组建癫痫患者自我管理干预小组（8名）：

成立以护士研究生为主导的医护协同团队，成员包括2名硕士研究生导师、2名主治医生、2名责任护士、2名在读全日制研究生。研究生导师负责监督和指导

癫痫自我管理项目工作的开展；在读研究生负责查阅、收集文献资料，制定自我管理的全程的干预方案；责任护士、研究生负责收集相关数据，落实护理措施为患者提供心理疏导，协助对患者进行管理和追踪随访；主治医生协助护士解决护理过程中遇到的问题。

②面对面交流评估及指导：

医生及护士与癫痫患者面对面介绍本次基于跨理论模型的实施自我管理行为教育项目的主要内容、目的、意义及参与规则，与患者建立良好合作的治疗性关系。结合跨理论模型（Trans Theoretical Model，TTM）初步评定患者自我管理的变化阶段，利用专业工具及量表于对患者进行常规评估，包括患者的行为改变阶段、知识掌握程度、生活质量、自我效能、用药依从性、心理情况等等，通过评估分析行为改变者的意愿及需求，根据患者的不同阶段为之提供有针对性的行为支持干预策略并每月进行电话随访，坚持自我管理的行为获得肯定；对于停留在前阶段或行为退回的患者，针对具体原因重新制定计划，从而帮助改变者建立健康行为。

前意向阶段：是指个体在未来6个月之内没有改变不良行为的意愿，该阶段个体的特点为不能/拒绝思考自己的不良行为所带来的后果，无改变意愿甚至对他人的健康建议存有抗拒心理，不会对过于简短的干预措施产生反应，是健康教育中的重点及难点。因此运用跨理论模型中的意识唤醒和生动解说方法转变患者态度，具体措施如下：

①通过图片、视频让患者回忆起疾病发作时的感受，了解患者目前疾病现状、对癫痫的认识情况、缺乏自我管理能力的原因，告知患者癫痫及自我管理的重要性并和对生活质量的影响。

②医护人员通过面对面知识讲座的形式介绍疾病相关知识，使患者了解癫痫的定义、病因、症状、类型及癫痫疾病的一般管理原则，转变患者的意识行为，针对原因进行具体分析，寻找行为改变的内在动机；

③向患者分发健康管理手册，使患者了解癫痫反复发作危害并唤起自我行为管理意识。

意向阶段：个体准备在6个月内改变不良行为，采纳健康的行为方式。该阶段个体的心理特点是体会到健康与行为方式有关，有一定的改变意愿，亦能够理解

改变可以带来良好的健康状况，但也顾虑到改变可能影响生活。因此，运用跨理论模型中的意识唤醒、自我再评价、环境再评价策略，分析、明确影响患者转变行为习惯的内在因素及外在因素，并做好相应的准备工作，具体措施如下：

①建立同伴协助微信群：邀请癫痫临床结局效果良好的患者分享经验，定期举办座谈会加强患者之间交流，再次通过知识的分享唤起患者的改变意识，认识到缺乏自我管理行为将会对身体及生活带来巨大的影响；

②具体分析患者当前认知与当前行为的不同之处，并对患者提出的积极态度给予肯定；

③专业人员陪同患者进行回忆分析，思考改变行为产生的阻碍因素及其具体原因；

④向患者发放癫痫日记记录本，为患者推荐疾病科普公众号及健康教育视频和网络，为患者行为转变提供科学、有效的辅助工具。

⑤干预人员需关注患者的心理状态，加强人文关怀，并让其学会自我欣赏、自我鼓励，同时从亲友处积极寻求帮助，树立战胜疾病的信心。

准备阶段：此期个体打算在1个月内采取行动，有行为动机，有一些改变或做了一定准备工作。通过前两次的行为阶段的转变，患者已经对自我管理行为有了较深的认识，并且打算在1个月内实施相关自我管理行为。制定计划以及能否积极实施是关键，干预人员利用跨理论模型中的自我解放与社会解放策略，与患者及家属一起制定切实可行的自我管理行为方案，具体措施如下：

①向强调接受系统的健康教育的益处，对患者正确的健康信念和态度作出积极正面的评价，并说明合理规律运动、遵医服药、及时参加健康教育并落到实处才能达到预期的效果；

②根据之前分析的影响患者的内在因素与外在因素与患者共同商量切实可行的行为转变计划，制定作息时间表、运动计划饮食记录等等；

③鼓励患者家属积极参与解决患者可能会遇到的心理问题和实际困难，让家属监督自我管理的实施情况；

④患者有疑问时请教医务人员，由专业人员向患者答疑解惑，并鼓励每位患者积极参与到自我管理行为的咨询当中；

⑤定期举办知识讲座：向患者介绍治疗癫痫的主要方法及补充代替疗法，在药物治疗中发挥积极作用改善癫痫发作，识别药物副作用及正确服药方法，提高患者依从性;教会患者识别癫痫发作影响因素及如何避免触发，减少癫痫发作及并发症发生。

行动阶段：此期个体行为已发生改变，但少于6个月，时间尚短，容易故态复萌。患者体会到进行自我管理行为所带来的益处，虽有自我管理行为的实施但尚不规律，持续的次数和时间未达到要求，自我管理行为的维持尚未超过6个月。此阶段患者能否正确实施自我管理行为并长期规律进行是关键。因此医护人员利用跨理论模型中的帮助关系、反条件化、强化管理、刺激控制策略进行干预，具体如下：

①建立癫痫日记，详细记录患者癫痫发作的类型、频率、程度、时长等，并记录与癫痫发作可能的原因以增强转变行为可能性。

②以科学知识和同伴成功经验为前提，根据目前癫痫发作情况及家属的反馈情况，结合他人成功经验及时调整计划；同时分析患者未成功保持的原因，及时给予健康教育和改进措施。

③鼓励患者克服进行自我管理行为过程中的困难，制定奖惩制度，动员家属给予帮助，对于保持良好的患者家属给予物质或精神奖励，并鼓励其继续坚持正确的自我管理行为。

④建立同阶段病友团体，互相监督与学习，共同完成自我管理行为并长期坚持;

⑤定期举办知识讲座：分享个人和社会的关系，癫痫如何影响个人生活和职业，让患有慢性疾病的患者生活正常化，通过减少对活动的不必要限制、建立和维持社会联系、减少孤立、立法和残疾人权利来减少耻辱感，追求积极的生活方式和独立生活。

维持阶段：此期个体行为改变并超过6个月 ，且已成为生活习惯，避免退回。此时向患者强调坚持长期规律执行自我管行为是关键。此阶段主要强化管理，避免健康行为的退回。因此，采用跨理论模型中的强化管理和避免退回策略进行干预，具体措施如下：

①患者实施自我管理行为前后的情况进行比较，激发其坚持自我管理行为的积极性;

②建立微信公众号，定期推送相关知识，并为患者提供咨询服务，解答患者疑问；

③通过电话随访、家属及病友监督等方式避免其自我管理行为的消退，若发生退回，及时寻找存在原因并予以解决。

质性研究

在实施完成年癫痫患者自我管理干预后，从干预组中随机选取 10名研究对象进行相关研究的质性访谈，选取标准为：

①完成全部阶段干预；

②愿意接受采访。

10名访谈对象尽可能均衡其一般人口学特征，2名经培训的调查员，按照拟定的访谈问题清单（见表 3），以一对一的访谈形式收集资料。所有的访谈都在征得访谈对象的同意后进行录音记录。所有录音都逐字逐句转录为文稿，再综合10名被访者资料，就每个问题的回答应用内容分析法进行分析和解释。

（3）研究工具

1） 问卷/量表的评估

①一般资料调查表：包含研究对象性别、年龄、婚姻情况、受教育水平等；抽烟、饮酒、睡眠、体育锻炼等等生活方式和习惯；癫痫疾病情况：病程、治疗方式、并发症、家族史、发作次数等；自评健康状况与改变意愿等。

②癫痫患者自我管理量表（Epilepsy Self-Management Scale, ESMS）：

是一种常用且有效的患者自我报告的38项量表，主要是为了评估癫痫患者自我管理实践的频率。它包含5个子量表，包括药物管理领域（即良好的药物依从性10项)，信息管理（即保持良好的癫痫记录8项)，安全管理（即避免饮酒8项)，癫痫发作管理（如癫痫发作比平时多立即联系医生6项)，生活方式管理（即压力管理6项)。本次研究应用中文版成人癫痫患者自我管理量（C-ESMS），各维度Cronbach，s alpha系数为：0.784-0.845，总量表Cronbach，s alpha系数为0.848。

③癫痫患者生活质量量表（QOLIE-31）：由克莱默[41]等人编制，是用于检查患者过去4周的生活并评估癫痫相关问题的治疗效果和患者总体健康状况的一种

自我报告结果工具。该问卷包含31个项目，这些项目被分成七个分量表，包括:发作担忧、生活质量、情绪健康、精力/疲劳、认知功能、药物效应和社会功能，子量表得分是根据每个子量表项目的平均值计算的，而总分是根据每个子量表得分的平均值与指定权重的乘积计算的。问卷采用李克特6分制，1为“所有的时间”，6为“没有任何时间”，问卷分数在0到100之间，分数越高，生活质量越好。刘雪琴[42]等人根据我国国情与文化差异，翻译修订了中文版QOLIE-31，并用于我国癫痫人群检测其信度、效度，该量表总的Cronbach’s alph α系数为0.9，分量表Cronbach’s alph α系数为0.58-0.88，具有较好的普适性，已被广泛用于评估我国患者生活状况。

④癫痫复发情况：制作癫痫发作情况记录本，由患者进行自我报告，报告内容包括发作时间、发作程度、应对情况等等。

2) 各量表的测量时间、次数及干预地点选择 ：

①一般情况调查表干预前测量；自我管理行为量表、癫痫生活质量量表、用药依从性量表于干预前、干预第1、3、6个月分别测量（分别记为：T0、T1、T3、T6；

4.质量控制

（1）正式调查前选取10名患者进行为期 3周的预实验，依据预实验反馈情况对患者干预方案进行修改整理完善，预实验所收集的资料未纳入研究数据中。

（2）调查前，对参与课题研究的调查员集中进行培训统一指导语和评判标准，尽可能减少偏倚。每次调查工作均由2名调查员共同完成。

（3）在选择研究对象的过程中，使用分层随机化严格把握两组患者的匹配原则，确保患者具有良好的可比性。在实施干预前取得患者所在医院的配合与支持并做好宣传工作，提高患者的积极性，确保研究的顺利进行。

（4）在一般情况调查时要求患者填写准确的基本信息，当研究过程中出现电话无法联系的情况，根据家庭住址寻找并及时更新联系方式。

（5）在正式访谈前进行自我反思避免用自身的观点与经历等引导受访者回答，提高访谈内容的可信性、可靠性。

5.统计方法

采用 SPSS18.0 处理分析数据 ，连续性资料用 $\bar{x}\pm s$进行展示，若服从正态分布采用两独立样本t检验， 若非正态采用非参数检验中wilcoxon(W)检验；分类资料采用计数和百分比进行展示，采用 $\chi 2$，校正 $\chi 2$，Fisher 精确检验进行评估；两组自我管理行为得分、生活质量评分、药物依从性、癫痫发作次数采用重复测量的方差分析，组内各时间点的比较采用 Bonferroni 法/Turkey法；癫痫知识两组组间比较采用非参数检验中的 wilcoxon(W)检验。

质性访谈结束后 24 小时内对录音资料进行逐字转录并与收集到的非语言资料进行整合。将文字资料返回受访者处确认转录的真实性。然后导入 Nvivo11 软件，以便数据分析。采用内容分析法对收集的资料进行分析：反复阅读所有原始资料，沉浸在资料中，对资料产生整体感；标注资料中的重要思想和概念，析取有重要意义的陈述语句；对反复出现的观点进行编码；将编码后的观点汇集；然后将相似或相关的编码归类形成主题和亚主题。

6.伦理原则

本研究方案实施前需通过遵义医科大学附属医院伦理委员会审议并批准实施，在研究进行期间，研究方案的任何修改均应经伦理委员会批准后方可执行。

(1)遵循知情同意原则：在开展研究前告知患者及家属本研究的研究目的、方法及研究过程，征得患者同意后方签署患者知情同意书，研究期间患者可随时提出退出研究，并且保证不影响患者的相关治疗及护理。

(2)遵循保密原则：研究过程中对患者填写的所有资料及个人信息进行严格保密，所有资料仅用于学术研究，必要时在研究结束之后进行资料销毁。

(3)遵循有益原则：本研究的研究目的是提高成年癫痫患者的自我管理能力，患者接受的健康教育、方法指导、电话随访、等有利于患者疾病的治疗。

(4)遵循公平原则：对干预组和对照组研究对象发放同样自我管理宣传的资料、对患者提出的问题给予满意答复。

7.本研究课题的创新之处

（1）理论上：本研究基于文献及理论著作分析、专家建议制定网络认知行为干预方案，与以往研究制定的认知行为干预方案相比，具有一定的科学性和可行性；

（2）方法上：本研究将量表测评与质性访谈相结合，两种研究结果相互印证、相互 补充，弥补了单一研究结果解释的局限性。

（3）技术上：本研究依托钉钉视频会议与微信群相结合的形式进行行为转变教育治疗，与现今在我国推行的“互联网＋医疗健康”的服务模式相契合

知情同意书

方案名称：基于TTM理论对成年癫痫患者自我管理的影响研究

方案版本号：V1.0，2022年06月20日

研究机构：遵义医科大学护理学院

主要研究者：李彩

您将被邀请参加一项临床研究，本知情同意书提供给您一些信息以帮助您决定是否参加此项临床研究。请您仔细阅读，如有任何疑问请向负责该项研究的研究者提出。本次研究经本机构生物医学研究伦理审查委员会审查通过。

研究目的：

①探讨基于TTM理论制定成年EP患者自我管理的方案比传统策略指导下的EP慢病管理是否具有优越性；

②探讨该方案对提升EP患者生活质量，改善不良心理状况，提高疾病自我管理能力的作用。

研究过程：通过随机对照试验方法开展自我管理干预，根据纳入排除标准选择遵义市成年癫痫患者，将其随机分为试验组和对照组。试验组除接受常规护理外还接受个性化自我管理方案与计划拟定，对照组仅接受常规健康教育，干预周期为6个月，干预前、干预后1、3、6个月照顾者进行量表自评，检验干预是否提高患者自我管理能力、减少癫痫发作频率、提高用药依从性、改善生活质量。

在本研究中您需要做什么？

配合接受相关知识教育，并在日常生活中执行相关行为。

风险与不适：

对于您来说，与我们进行沟通、交谈可能会有些心理不适。

由于本研究仅涉及收集病人或正常人病史、数据及流行病学调查等，因此该项研究不存在任何损害；如发生与该项临床研究相关的损害时，您可以获得免费治疗和/或根据中国法律获得相应的补偿。

参与本研究有何受益？

通过对您的信息资料进行研究，可能为疾病的研究提供有益的信息。

参与本研究的费用：

参与本研究将通过对您的医疗信息资料进行研究，相关费用不由您承担。

隐私问题：

如果您决定参加本项研究，您参加试验及在试验中的个人资料均属保密。负责研究医师及其他研究人员将使用您的医疗信息进行研究。这些信息可能包括您的姓名（将采用编号形式进行处理）、地址、电话号码、病史及在您研究来访时得到的信息。您的档案将保存在有锁的档案柜中，仅供研究人员查阅。为确保研究按照规定进行，必要时，政府管理部门或伦理审查委员会的成员按规定可以在研究单位查阅您的个人资料。这项研究结果发表时，将不会披露您个人的任何身份信息。

您是否可以退出本研究？

您参加本项研究是自愿的。您可以选择不参加本项研究，或者在任何时候申请退出研究，您不会受到任何不公平的待遇，您的数据将不纳入研究结果，您的任何医疗待遇与权益不会因此而受到影响。

如果您需要其他治疗，或者您没有遵守研究计划，或者发生了与研究相关的损伤或者有任何其他原因，研究医师可以终止您继续参与本项研究。

您可随时了解与本研究相关的信息资料和研究进展，如果您有与本研究有关的问题，或您在研究过程中发生了任何不适与损伤，或有关于本项研究参加者权益方面的问题您可以通过________（*电话号码*）与__李彩__（*研究者或有关人员姓名*）联系。

知情同意书签署页

我已经阅读了本知情同意书。

我有机会提问而且所有问题均已得到解答。

我理解参加本项研究是自愿的。

我可以选择不参加本项研究，或者在任何时候通知研究者后退出而不会遭到歧视或报复，我的任何医疗待遇与权益不会因此而受到影响。

如果我需要其他治疗，或者我没有遵守研究计划，或者发生了与研究相关的损伤或者有任何其他原因，研究医师可以终止我继续参与本项研究。

我将收到一份签过字的“知情同意书”副本。

受试者签名：____________________ 受试者联系电话：____________

日期：______年_______月_______日

法定代理人签名： 与受试者关系：______________

联系电话：____________________

日期：______年_______月_______日

监护人签名： 与受试者关系：______________

联系电话：____________________

日期：______年_______月_______日

我确认已向受试者解释了本研究的详细情况，包括其权利以及可能的受益和风险，并给其一份签署过的知情同意书副本。

研究者姓名：_________________

研究者签名：_________________

日期：_______年________月________日

（注：如果受试者不识字时尚需见证人签名，如果受试者无行为能力时则需代理人或监护人签名）

遵义医科大学附属医院生物医学研究伦理委员会办公室联系电话：0851-28608776
