# Supplementary material for: Effects of the transtheoretical model-based self-management program on behavioral change in persons with epilepsy: Study protocol for a randomized controlled trial
Source: PLoS One. 2024 Nov 25;19(11):e0305547. doi: 10.1371/journal.pone.0305547 (PMC11588227; doi:10.1371/journal.pone.0305547)
Supplement: S2 File — (PDF) [file pone.0305547.s003.pdf]

Biomedical Research Ethics Committee of Zunyi Medical University Hospital

Ethical review approval

Ethical Review Approval Letter No.: KLLY-2022-062

Acceptance number	KLLY-2022-062		
project name	Study on the influence of TTM theory on self-management of adult epileptic patients		
Project source	Master's thesis opening project		
project leader	Li CAI	Master student	Huang Hao
The department of the project leader	School of nursing		
Review category	Initial review	Review method	Quick review
Review time	2022.12.31	Review the location	The Office of the Ethics Committee
audit-review file	1. Application for ethical review<br>2. Opening report (version date: 2022-06-30)<br>3. Informed Consent (Version: V1.0; dated: 2022-06-20)		

Review opinions:

According to the world medical association, the international medical science organization committee "international biomedical research ethics guidelines", "drug clinical trial quality management standard", "involving the human body," drug clinical trial ethical review guidelines " and other laws, regulations, rules, normative documents and international guidelines, the ethics committee

The staff will review and agree that the project will be conducted according to the scheduled protocol.

Please follow the EC approved protocol to protect the health and interests of the subjects.

1. If the project leader is changed during the study, the study protocol, informed consent form, case report form, questionnaire and recruitment will be conducted

For any amendment to the fundraising materials, please submit the amendment application for review;

2. Please timely inform us of Zunyi Medical University in accordance with the relevant laws and regulations and the safety event report plan in the research plan

The Biomedical Research Ethics Committee of the Affiliated Hospital submitted a written adverse event report;

3. The investigator did not follow the protocol to conduct the study, which may contribute negatively to the rights / health of the subjects and the scientific nature of the study

Ring, please submit the violation event report;

4. If the applicant suspends or terminates the clinical study early, please submit the suspension / suspension report in time;

5. At the end of the study, please submit the final report.

6. Please submit the application for continuous review at least one month prior to the expiration date.

Date of approval 2022.12.31	Date of failure of approval2024.12.30

Application form for ethical review of scientific research projects

Project Name: Study on the impact of TTM theory on self-management in adult epilepsy

project leader: Li CAI

Scientific Research project category:
National, provincial and ministerial level ☐ bureau level / university level other ☐☐☐☑

Department (major) of the hospital: Department of Neurology (nursing)

Date of filling in the form: December 27,2022

project name	Study on the influence of TTM theory on self-management in adult patients with epilepsy		
Project source			
Opening topic report version number	*V1.0*	Thesis proposal Version date	*2020.9.27*
informed consent version number	*V1.0*	informed consent Version date	*2020.9.27*
The sponsor or the project sponsor			
contacts	*Li CAI*	contact number	
Group leader unit			
Principal investigator of the team leader unit	*Li CAI, Huang Hao*		
Participate in the unit			
The hospital is responsible for the department	neurology		
The principal investigator of our hospital	*Li CAI, Huang Hao*		

1. Research information

- Scientific purpose:

① To explore whether the self-management program for adult EP patients based on TTM theory is superior to the chronic disease management of EP under the guidance of traditional strategies;

② Explore the role of this program in improving the quality of life of EP patients, improving the adverse psychological condition, and improving the ability of disease self-management.

Scheme design type

- ☑experimental study
    - ☐ Observational study: ☐ Retrospective analysis, ☐ Prospective study
    - ☐ Use of human biological specimens: ☐ Previous collection and preservation, ☐ research collection
- Possible adverse reactions and preventive measures (please specify):
- Special conditions required

☐ Intensive care, ☐ isolation zone, ☐ surgery, ☐ pediatric intensive care, ☐ intravenous infusion,

☐ Computed tomography, ☐ gynecology, ☐ gene therapy, ☐ controlled drugs (anesthetic / psychotropic drugs), ☐ Other (please specify):

____

- Study duration: from June 2022 to June 2024
- Additional study information:
    - ✧ Source of funds: ☐ enterprise, ☐ government, ☐ academic group, ☐, the unit, self-raised☑
    - ✧ Has the research project been rejected or rejected by other ethics committees?

      ☑ None, ☐ Please submit the relevant documents
    - ✧ Has the research project ever been suspended or terminated by other ethics committees?

☑None, ☐ Please submit the relevant documents

- ✧ Are there similar research projects in our department?☐ is, no☑
- ✧ In the current research projects, the number of projects is the same as the target diseases of this project: ____.
- ✧ Studies requires the use of human biological specimens:
  ☑deny,
  ☐ Fill in the following options
  - ➤ Biological specimens were collected: ☐ Yes, ☐ No
  - ➤ Using previously preserved biological specimens: ☐ Yes, ☐ No

- Recruitment of subjects:
  - ✧ ☐Who is responsible for recruiting: physician, ☐ investigator, ☐ research assistant, research nurse,☑
    ☐ Others: ____
  - ✧ Recruitment method: ☐ advertising, ☐ diagnosis and treatment process, database, ☐ intermediary, ☐ Others: _______☑
  - ✧ Recruitment population characteristics:

☐ Healthy person, ☐ patient (please describe): Adult epilepsy patients

Age range of subjects: _ 18 years ____ Subject sex: __ No special requirements _____________

☑Subjects (☐ Yes, no) with vulnerable groups: _______ (if so, please mark the vulnerable groups involved. Such as children / minors, adults with cognitive impairment or health status unable to give informed consent, employees or students of the sponsor / investigator, persons with education / low economic status, patients with end-stage disease, prisoners or reeducation through labor, pregnant women, etc.)

- ✧ Subject remuneration: ☐ Yes, No☑
  - ➤ Compensation amount: ()__________ can be completed
  - ➤ Payment method: ☐ According to the follow-up observation point,

☐ One-time payment according to the completed follow-up observation workload,

☐ Payment after completing all follow-up observations

- Process of informed consent:
  - ✧ Who obtains the informed consent: ☐ Doctor / Investigator, ☐ Doctor,
    ☑Investigator, ☐ Other: ________________
  - ✧ Location to obtain informed consent: ☐ private room / subject reception room, ☐ clinic, ward,☑
    ☐ Others: ___________________________
  - ✧ ☑Signature of informed consent: signature of subject, signature of ☐ legal representative and signature of ☐ guardian
- Exception to informed consent:
  ☑deny,
  ☐ Select the following options:
  - ✧ ☐ Apply for studies where informed consent is not available in an emergency:
    - ➢ The study population is in a life-threatening emergency that requires intervention soon after disease onset;
    - ➢ In this emergency, most patients are unable to give informed consent and have no time to find a legal representative;
  - ✧ ☐ Application for exemption from informed consent —— Use of medical records / biological specimens obtained from previous clinical diagnosis and treatment;
  - ✧ ☐ Application for exemption from informed consent —— for secondary use of study medical records / biological specimens;
  - ✧ ☐ Application for exemption of informed consent signature —— The signed informed consent form will pose an improper threat to the subject's privacy. The only record to contact the true identity of the subject and the study is the informed consent document, and the main risk comes from the disclosure of the subject's identity or personal privacy;

✧ ☐ Application for exemption from informed consent signature —— Study is not greater than the minimum risk to subjects, and in the same context, such as an interview study, email / telephone survey

2. Project researchers

1. Our hospital:

Principal investigator: Li CAI Title: Nurse

Department: Department of Neurology Tel:

attendee:

(1) Main contact person: Huang Hao. Professional Title: Deputy Chief physician

Department: Department of Neurology Tel:

(2) Other participants: (if the personnel of other departments are involved, please indicate their departments)

professional ranks and titles:

Department: division of labor

professional ranks and titles:

Department: division of labor

professional ranks and titles:

Department: division of labor

professional ranks and titles:

administrative or technical offices:

2. Other units (if involved, please fill in the following contents):

①

Principal Investigator: Professional Title:

②

Principal Investigator: Professional Title:

③

Principal Investigator: Professional Title:

Investigator Responsibility Statement	I will follow the study protocol and the requirements of the ethics committee		
Signed of the principal investigator		date	
Signature of department director		date	

Ethical application trial protocol

summary
Source of funding: This project was awarded to the National Natural Science Foundation of China (No.81760247; 82171450), The Youth Science and Technology Talents Training Program of The Education Department of Guizhou Province (No. KY (2017) 202), Neurology Graduate Workstation of Zunyi Medical College (No. GZZ2017004), supported by the Doctoral Research Fund of The Affiliated Hospital of Zunyi Medical University (Approval No.: (2016) 14) and Guizhou Provincial Science and Technology Fund [2019) No.1350],
Study name: Based on the impact of behavioral stage transition theory on self-management in adult epilepsy
Project study site: Affiliated Hospital of Zunyi Medical University
**Number of participants: 154 participants**
**Main person in charge: Li Cai**
Study time: August 2023, March 2024
**purpose:**<br>① Construct self-management intervention programs for adult epilepsy patients under the guidance of behavioral stage transition theory<br>② Validate the self-management intervention program for adult patients with epilepsy under the guidance of the behavioral stage transition theory<br>③ Investigate the effect evaluation of the self-management program in adult patients with epilepsy
**Study design: This study is a randomized, participant-blinded,**

controlled clinical trial.
**Research criteria:**<br><br>Inclusion criteria:<br><br>① Meet the diagnostic criteria for epilepsy published by the 2017<br><br>International Alliance against Epilepsis (ILAE 2017);<br><br>② 6 months and at least 1 seizure during the period;<br>③ Take at least one antiepileptic drug;<br>④ Age: 18 years old;<br>⑤ Primary school education or above;<br>⑥ Living in the city for a long time;<br>⑦ Can accept the intervention program, informed consent and voluntary participation.<br>Exclusion criteria:<br><br>① A history of brain surgery;<br>② Diagnosis of acute symptomatic seizures related to acute neurological disease or substance abuse;<br>③ Combined with other neurological or mental diseases, mental and personality disorders, unable to communicate normally;<br>④ Serious heart, liver, kidney and other physical diseases, combined with malignant lesions, progressive or degenerative diseases;<br>⑤ Is participating in other clinical subjects.<br>Criteria for shedding:<br>① Transfer to another hospital for treatment during the study;<br>② Did to complete all intervention and data collectors;<br>③ Voluntary withdrawal from the investigator.
Sample size: In this study, G-power3.1 software was used to calculate the sample size, and the main statistical analysis method was T test, and N1 / N2=1, α =0.05, effectsize = 0.05, effectsize = 0.05, Table was two-sided, Power=0.80, and the total sample size was 128 cases. The loss to follow-up probably increased by 20%, and the calculated sample size was 160 patients.

Study protocol construction

1. Preliminary construction of self-management intervention programs for epilepsy patients

Systematic review of relevant literature and guidelines at home and abroad, screened and extracted relevant indicators and influencing factors of self-management of epilepsy at home and abroad, combined previous literature research results and relevant research theories with the disease characteristics of epilepsy patients, and preliminarily constructed the first draft of self-management intervention program.

(1) Systematic literature review of the influencing factors, assessment tools of epilepsy self-management and intervention programs

We will develop the search form according to the characteristics of each database, use Boolean logical words to connect each search term, and extract the content related to the self-management of epilepsy, Search the original studies, include the exclusion criteria:

Inclusion criteria: ① Study subjects were 18-years-old adult patients with epilepsy;

② The research content involved the epilepsy self-management and its influencing factors and intervention measures;

③ The language is in either Chinese or English.

Exclusion criteria: ① Review;

② No access to the full text;

③ Repeat for the published literature.

(2) screening technique

Two researchers will complete the literature screening process independently, first read the article title and abstract, exclude the significant unrelated literature, further read the full text,

eliminate the literature that does not meet the inclusion criteria, if the information is incomplete, contact the author to obtain, if in case of disagreement, discuss with the team leader.

The Chinese version of the Cochrane included RCT literature quality evaluation (Risk Bias assessment tool) was used to evaluate the evidence grade of the original study and include the original study with low risk of offset. Use Excel 2016 to input basic literature information and the content, observation indicators of epilepsy self-management intervention and influencing factors.

(3) RCT construct intervention program with low risk and good outcomes

Relevant data were summarized through Excel 2016 to summarize the contents of the interventionists, intervention methods and intervention effects of epilepsy self-management, and to extract the original study with good intervention outcomes to form the first draft of the intervention plan of this study.

2. Revision of the self-management intervention program for patients with epilepsy

Medical experts in the field of epilepsy were invited to carry out expert consultation, discuss the scientificity and feasibility of the preliminary constructed self-management intervention program for epilepsy patients through the Delphi method, and revise and improve it again combined with the feedback given by experts to finally form the intervention program.

(1) Inclusion criteria for experts

Inclusion criteria for medical experts: ① Master's degree or above; ② Deputy senior or above title;

③ At least 10 years or work in the field of neurology;

④ Voluntary participation in the expert consultation in this study.

Inclusion criteria for nursing experts: ① Bachelor degree or above;

② Deputy senior or above title;

③ At least 5 years of work in the field of neurology;

④ Voluntary participation in the expert consultation in this study.

(2) Determine the number of experts

Based on the homogeneity of the expert work field, 10 experts were selected for consultation.

(3) Design of the expert consultation form

Include 3 parts:

① Letter to the expert (explaining the purpose and significance of the study, filling in the form, the expected time of completion, giving thanks to the expert, etc.);

② Basic information investigation of experts (basic information of experts, self-evaluation of familiarity with the consultation content and judgment basis, etc.);

③ Main content of the consultation (content bar of each item in the intervention program, scoring bar of item importance, modified opinion bar, new entry bar, etc.).

(4) Implementation steps of expert consultation

Brief the research purpose, significance and task to the experts. After obtaining the consent of the experts, use the first questionnaire star distribution consultation form, and it will be collected in time. The opinions of the experts will be sorted out, and then the second expert consultation will be conducted. When the

opinions are consistent, the consultation will be stopped to form the final epilepsy self-management intervention program.

(5) Statistical analysis

Data were derived from the questionnaire star platform, and the statistical analysis was performed using SPSS 18.0:

① General expert data: measurement data are expressed by mean and standard deviation, and counting data are expressed by frequency and percentage;

② Experts' enthusiasm to use the recovery rate of the questionnaire expressed;

③ The degree of expert authority is expressed by the authority coefficient (Cr);

④ The concentration of expert opinions is expressed by the mean of items and the frequency of full marks;

⑤ The degree of expert opinion coordination is expressed by the coefficient of variation and Kendall harmony coefficient (Kendall's W).

3. An empirical study of a self-management intervention program for epilepsy patients based on the theory of behavioral stage transition

(1) subject investigated

The epilepsy patients who presented to the Outpatient Department of Neurology Department of the Affiliated Hospital of Zunyi Medical University met the inclusion criteria during the study time as the study subjects. Baseline data of all patients were evaluated before the intervention, Including general information, quality of life, self-management, etc., Divided into the experimental group and the control group according to the random number table method, In a six-month intervention study, The control group implemented the conventional epilepsy health education method, The trial group

conducted a self-management behavioral intervention using a health education model based on the transtheoretical model, Before intervention, intervention 1,3 and 6 months, The stage of behavior change and the effect of self-management behavior intervention were assessed by the epilepsy self-management scale (ESMS), epilepsy quality of life scale (QOLIE-31), the number of seizures, Then evaluate the effect of self-management intervention in adult epilepsy patients based on TTM theory.

(2) The research plan

Quantitative study

Before the intervention, face-to-face questionnaire survey was adopted. For the research subjects who met the qualified standards, the qualified investigators will issue paper questionnaire or electronic questionnaire on the spot, and the respondents will fill in the questionnaire by themselves. For those who were unable to complete the questionnaire independently due to limited educational level, the investigators will read the questionnaire and the respondents will make self-evaluation. All the questionnaires were collected on the spot to ensure their authenticity and validity. This study was single-blind, that is, the study subjects did not know the specific grouping situation. In this study, the subjects were numbered in the order of the study, following a 1:1 ratio, stratified randomization (12 or <12 seizures per year), and randomization with the assistance of the doctor, designed to ensure equal access of patients to each group to match the general demographic characteristics of the two groups so as to equally distribute the severity of the seizures between the two intervention groups and reduce the possibility of bias.

1) Control group: routine epilepsy health education

Time: routine treatment and health consultation for 6 months, outpatient visit once / month, 30 minutes / time.

content:

① Explain the relevant knowledge of the disease to the patients, distribute the "epilepsy Disease Publicity and Education Manual", and answer the patients' questions;

② Visit on time every month and visit time if you have any problems.

2) **Test group: Self-management health education based on the transtheoretical model (TTM)**

Time: 6-month intervention, 1 / month, 6 visits, 30-60 minutes / time; outpatient visit / month, 30 minutes, telephone follow-up 1 / month, 20 minutes, 6 times.

Form: Based on the theory of TTM, conduct the same health education intervention as the control group, in addition to the content of the control group, conduct personalized health education and self-management plan, and monthly telephone follow-up.

content:

① Establish a self-management intervention team for epilepsy patients (8 patients):

A medical collaborative team led by nurse graduate students was established, with members including 2 postgraduate supervisors, 2 attending doctors, 2 responsible nurses and 2 full-time graduate students. The graduate supervisor is responsible for supervising and guiding the epilepsy self-management project; the graduate students are responsible for consulting and collecting literature and formulating the intervention program of self-management; the responsible nurses and graduate students are responsible for collecting relevant data, implementing nursing measures to provide

psychological counseling for patients, assisting in the management and follow-up of patients; the attending doctors assist nurses to solve the problems encountered in the nursing process.

② Face-to-face communication assessment and guidance:

Doctors and nurses and patients with epilepsy face to face to introduce the main content, purpose, significance and participation rules of the self-management behavior education project based on the intertheoretical model, and establish a good cooperative therapeutic relationship with patients. Combined with the cross-theoretical model (Trans Theoretical Model, TTM) initial stages of change in patient self-management, Using professional tools and scales for the routine assessment of patients, Including the stages of behavior change, knowledge mastery, quality of life, self-efficacy, medication compliance, psychological conditions, and so on, By assessing and analyzing the wishes and needs of behavior alters, Provide targeted behavioral support intervention strategies and monthly telephone follow-up, The behavior of maintaining self-management is recognized; For patients who stayed in the previous phase or behavior behavior, Replanning for specific reasons, Thus helping alters to establish health behavior.

Before intention stage: refers to the individual in the next six months without change the will of bad behavior, the characteristics of the individual can not / refused to think about the consequences of their bad behavior, no change will even to others health advice have resist psychological, not respond to too brief interventions, is the key and difficulty in health education. Therefore, the consciousness awakening and vivid explanation methods in the transtheoretical model are used to change the patients' attitudes. The specific measures are as follows:

① Through pictures and videos, let the patients recall the feelings of the disease, understand the current situation of the disease, the understanding of epilepsy, the reasons for the lack of self-management ability, and inform the importance of epilepsy and self-management and the impact on the quality of life.

② Medical staff introduce the relevant knowledge of the disease in the form of face-to-face knowledge lectures, so that patients understand the definition, etiology, symptoms, types and general management principles of epilepsy, change the conscious behavior of patients, make specific analysis of the causes, and find the internal motivation of behavior change;

③ distribute health management manuals to patients to understand the dangers of recurrent seizures and raise awareness of self-behavior management.

Intention phase: Individuals are prepared to change bad behavior and adopt healthy behavior within 6 months. The psychological characteristics of individuals at this stage are that they realize that health is related to behavior, and have a certain willingness to change. They can also understand that change can bring good health status, but they also worry that change may affect life. Therefore, the strategies of consciousness awakening, self-evaluation and environmental reevaluation in the transtheoretical model are used to analyze and clarify the internal factors and external factors affecting patients' change of behavior habits, and make corresponding preparations. The specific measures are as follows:

① Establish peer assistance WeChat group: invite patients with good clinical outcomes of epilepsy to share their experience, hold regular forums to strengthen the communication between patients, arouse patients' awareness of change through the sharing of knowledge again,

and realize that the lack of self-management behavior will have a huge impact on the body and life;

② Analyze the differences between patients' current cognition and current behavior specifically, and affirm the positive attitude of patients;

③ Professionals accompany patients to recall analysis, thinking about the hindering factors of behavior change and the specific reasons;

④ Issue epilepsy diary books to patients, recommend disease popularization public science accounts and health education videos and networks for patients, and provide scientific and effective auxiliary tools for patients' behavior change.

⑤ Interventionist personnel should pay attention to the psychological state of patients, strengthen humanistic care, and let them learn to self-appreciation, self-encouragement, at the same time actively seek help from relatives and friends, establish confidence to overcome the disease.

Preparation stage: During this period, individuals intend to take action within 1 month, are motivated to behave, have some changes or do some preparatory work. Through the first two behavioral stages, patients already have a deep understanding of self-management behavior and intend to implement relevant self-management behavior within 1 month. The key is that the intervention personnel use the self-liberation and social liberation strategies in the transtheoretical model to develop feasible self-management behavior programs together with the patients and their families. The specific measures are as follows:

① To emphasize the benefits of receiving systematic health education, make a positive evaluation of patients' correct health beliefs and attitudes, and explain that reasonable and regular exercise, medical

compliance, timely participation in health education and implementation to achieve the expected results;

② According to the previous analysis of internal factors and external factors affecting patients, discuss feasible behavior change plan with patients, formulate work schedule, exercise plan and diet records, etc.;

③ Encourage the patient's family members to actively participate in solving the psychological problems and practical difficulties that the patient may encounter, and let the family members supervise the implementation of self-management;

④ When patients have questions, consult medical staff, by professionals to answer questions to patients, and encourage each patient to actively participate in the consultation of self-management behavior;

⑤ Regular knowledge lectures: introduce the main methods of epilepsy treatment and complementary therapy to patients, play an positive role in drug treatment to improve seizures, identify drug side effects and correct medication methods, improve patient compliance; teach patients to identify the influencing factors of seizures and how to avoid triggering, reduce seizures and complications.

Action stage: the individual behavior has changed, but less than 6 months, the time is still short, and it is easy to relapse. Patients realize the benefits of self-management behavior. Although the implementation of self-management behavior is not regular, the duration and time have not meet the requirements, and the maintenance of self-management behavior has not exceeded 6 months. At this stage, whether patients can correctly implement self-management behavior and long-term rule is the key. Therefore, medical staff use the help relationship, anti-conditioning, strengthening management and stimulation control strategies to intervene, as follows:

① Establish an epilepsy diary, detail the type, frequency, degree and duration of the seizure, and record the possible cause of the seizure to enhance the possibility of transition behavior.
② On the premise of scientific knowledge and successful experience of peers, adjust the plan according to the current seizure situation and feedback of family members and the successful experience of others; analyze the reasons for unsuccessful maintenance, and provide timely health education and improvement measures.
③ Encourage patients to overcome the difficulties in the process of self-management behavior, formulate a reward and punishment system, mobilize family members to give help, give material or spiritual rewards to patients' family members who keep good, and encourage them to continue to adhere to the correct self-management behavior.
④ Establish a group of patients at the same stage, supervise and learn from each other, complete self-management behavior together and adhere to it for a long time;
⑤ Regular knowledge lecture: share the relationship between individual and society, how epilepsy affect personal life and occupation, make patients with chronic disease life normalization, by reducing unnecessary restrictions on activities, establish and maintain social ties, reduce isolation, legislation and the rights of the disabled to reduce stigma, the pursuit of positive lifestyle and independent life.
Maintenance phase: this period of individual behavior change and more than 6 months, and has become a living habit, avoid return. At this time, it is the key to emphasize to the long-term rules of self-management behavior. At this stage, we should mainly strengthen the management to avoid the return of health behaviors. Therefore, the intensive management and return avoidance strategy in the transtheoretical model are adopted as follows:

① Compare the situation of patients before and after the implementation of self-management behavior to stimulate their enthusiasm to adhere to self-management behavior;
② Establish a wechat public account, regularly push relevant knowledge, and provide consultation services for patients, and answer patients' questions;
③ Avoid the extinction of self-management behavior through telephone follow-up, supervision of family members and patients. If they return, find the reasons in time and solve them.

qualitative research

After the implementation of the self-management intervention for the annual epilepsy patients, 10 study subjects were randomly selected from the intervention group for the qualitative interviews of the relevant study. The selection criteria were:
① Complete all stages of the intervention;
② Willing to be interviewed.
Ten interviewees were as balanced as possible, and two trained investigators collected data through one-to-one interviews according to the proposed list of interview questions (see Table 3). All the interviews were audio-recorded after obtaining the consent of the interviewees. All the recordings were transcribed word by word, and the data of 10 respondents were analyzed and interpreted by analyzing the answers to each question.

(3) Research tools
1) Assessment of the questionnaire / scale
① General data questionnaire: including gender, age, marital status, education level, etc.; smoking, drinking, sleeping, physical exercise and other lifestyles and habits; epilepsy diseases: course, treatment,

complications, family history, attacks; self-rated health status and willingness to change, etc.

② The Self-Management Scale for Epilepsy Patients (Epilepsy Self-Management Scale, ESMS):

Is a commonly used and validated patient self-reported 38-item scale, primarily designed to assess the frequency of self-management practices in patients with epilepsy. It contains five scales, including drug management (i. e., good drug compliance 10), information management (i. e. keep good epilepsy record 8), safety management (i. e., avoid drinking 8), seizure management (e. g. seizures more than usual immediately contact doctor 6), lifestyle management (i. e., stress management 6). In this study, the Chinese version of self-management (C-ESMS), Cronbach, s alpha coefficient is 0.784-0.845, total table Cronbach, and s alpha coefficient is 0.848.

③ Quality of Life Scale for Epilepsy (QOLIE-31): developed by Cramer [41] et al., is a self-reported outcome tool used to examine the past 4 weeks and evaluate the treatment effects of epilepsy-related problems and the overall health of the patient. The questionnaire contains 31 items divided into seven subscales, including episode worry, quality of life, emotional health, energy / fatigue, cognitive function, drug effects, and social function, the subscale score was calculated from the average of each subscale item, while the total score was calculated from the product of the average of each subscale score and the specified weights. The questionnaire used a Likert 6-point scale, 1 is "all time", 6 is "no any time", questionnaire scores between 0 and 100, the higher the score, the better the quality of life. According to China's national conditions and cultural differences, [42] et al. translated and revised the Chinese version of QOLIE-31, and used it to detect the reliability and validity of the epilepsy population in China. The total Cronbach's

alph α coefficient of this scale is 0.9, and the Cronbach's alph α coefficient of the subscale is 0.58-0.88, which has good universality and has been widely used to evaluate the living conditions of patients in China.

④ Epilepsy recurrence: Make a seizure situation record book, and the patient will self-report, and the report content includes the attack time, seizure degree, response and so on.

2) The time and frequency of each scale:

① General Situation questionnaire measured before intervention; Self-management behavior scale, Epilepsy quality of life scale and medication compliance scale were measured before intervention and at the 1,3 and 6 months of intervention (recorded as T0, T1, T3 and T6;

4. Quality control

(1) Before the formal investigation, 10 patients were selected for the 3-week pre-experiment, and the patient intervention program was modified and improved according to the feedback of the pre-experiment. The data collected by the pre-experiment were not included in the study data.

(2) Before the investigation, the investigators involved in the research should be trained with unified guidance and evaluation criteria to reduce bias as far as possible. Each investigation was completed by two investigators.

(3) In the process of selecting research subjects, stratified randomization was used to strictly grasp the matching principle of the two groups of patients to ensure the good comparability of patients. Before the implementation of the intervention, the cooperation and support of the hospital where the patients work and the publicity should be done to improve the enthusiasm of the patients and ensure the smooth progress of the study.

(4) In the general situation investigation, patients are required to fill in accurate basic information. When they cannot be contacted by phone during the study, they should find and update their contact information in time according to their home address.

(5) Conduct self-reflection before the formal interview to avoid guiding the interviewees to answer with their own views and experiences, so as to improve the credibility and reliability of the interview content.

5. Statistical methods

Analysis of the data were processed using SPSS18.0, The continuity data are presented with $x \pm s$, If two independent t-test of samples has followed the normal distribution, If non-normal, use the wilcoxon (W) test in the non-parametric test; Categorical data were presented using counts and percentage, Using the $\chi 2$, Correction for the $\chi 2$, Fisher Accurate test for evaluation; Two groups of self-management behavior scores, quality of life scores, medication adherence, number of seizures were analyzed using repeated measures, The comparison of each time point in the group was made by the Bonferroni method / Turkey method; The comparison of epilepsy knowledge between the two groups used the wilcoxon (W) test in the non-parametric test.

The audio recordings were transcribed verbatim within 24 hours after the qualitative interview and integrated with the collected non-verbal data. The written data were returned to the respondents to confirm the authenticity of the transcription. The Nvivo11 software was then imported, allowing for data analysis. The content analysis method is used to analyze the collected data: read all the original materials repeatedly, immerse in the data, produce the overall sense of the data; mark the important ideas and concepts in the data, analyze the important statements; code the recurring views; collect

the views after coding; and then classify the similar or related codes to form themes and subthemes.

6. Ethical principles

The study protocol should be reviewed and approved by the Ethics Committee of the Affiliated Hospital of Zunyi Medical University Administration, during the study, any modification to the protocol should be performed after ethics committee approval.

(1) Follow the principle of informed consent: inform the patient and his family members of the research purpose, method and process of the study before the study, sign the informed consent of the patient, the patient can withdraw from the study at any time during the study, and ensure that the relevant treatment and care of the patient will not be affected.

(2) Follow the principle of confidentiality: In the course of the study, all data and personal information filled in by patients shall be kept strictly confidential. All data shall only be used for academic research, and the data shall be destroyed after the end of the study if necessary.

(3) Follow the principle of benefit: The purpose of this study is to improve the self-management ability of adult patients with epilepsy, and to improve the health education, method guidance, telephone follow-up, and other favorable treatment of patients' diseases.

(4) Follow the principle of fairness: give the research subjects of the intervention group and the control group the same self-management publicity materials, and give satisfactory answers to the questions raised by the patients.

7. The innovation of this research topic

(1) Theoretically: This study is based on the analysis of literature and theoretical works, and the formulation of online cognitive behavior intervention program suggested by experts. Compared with the cognitive behavior intervention program formulated in previous studies, it is scientific and feasible;

(2) Methodology: This study combines the scale evaluation with the qualitative interview, and the two study results confirm and complement each other, making up for the limitation of the interpretation of the single study results.

(3) Technically: This study relies on the combination of nail video conference and wechat group to conduct behavior transformation education and treatment, which is in line with the service mode of "Internet + medical care" implemented in China

informed consent

Protocol name: Study on the impact of TTM theory on self-management in adult epilepsy

V1.0, June 20,2022

Research institution: School of Nursing, Zunyi Medical University

Principal investigator: Li Cai

You will be invited to participate in a clinical study, and this informed consent form gives you some information to help you decide whether to participate in this clinical study. Please read it carefully and ask any questions to the investigator responsible for the study. This study was reviewed and approved by the Biomedical Research Ethics Review Committee of our institution.

purpose of research:

① To explore whether the self-management program for adult EP patients based on TTM theory is superior to the chronic disease management of EP under the guidance of traditional strategies;

② Explore the role of this program in improving the quality of life of EP patients, improving the adverse psychological condition, and improving the ability of disease self-management.

Study process: Self-management intervention was carried out through the randomized controlled trial method, and adult epilepsy patients in Zunyi city were selected according to the inclusion and exclusion criteria and randomly divided into trial group and control group. Test group in addition to accept routine care, personalized self management plan and plan, the control group only accept routine health education, intervention cycle for 6 months, intervention and 1,3,6 months after the caregivers scale self-evaluation, test whether the intervention to improve patients' self-management ability, reduce

the frequency of seizures, improve medication compliance, improve the quality of life.

What do you need to do in this study?

Cooperate to receive relevant knowledge education, and perform relevant behaviors in daily life.

Risk and discomfort:

For you, there may be some psychological discomfort to communicate and talk with us.

Since the study only involves the collection of patient or normal medical history, data and epidemiological investigation, the study has no damage; you may receive free treatment and / or, corresponding compensation under Chinese law.

What are the benefits of participating in this study?

By studying your information, you may provide useful information for the study of the disease.

Cost of participation in this study:

Participation in this study will be based on your medical information and the costs are not borne by you.

privacy problem:

If you decide to participate in this study, your participation in the trial and your personal data during the trial are kept confidential. The responsible study physician and other investigators will use your medical information for the research. This information may include your name (which will be processed in a numbered form), address, telephone number, medical history, and information received during your study visit. Your file will be kept in a locked file cabinet for researcher access only. To ensure that the study is conducted in accordance with the regulations, members of the government administration or the ethics review committee can access your personal data at the study site if necessary. No any of your

personal identifying information will be disclosed at the time of publication of this study results.

Can you withdraw from this study?

Your participation in this study is voluntary. You may choose not to participate in this study or apply to withdraw from the study at any time, you will not be treated unfairly, your data will not be included in the study results, and any medical treatment and interests will not be affected.

If you need other treatment, or you have not comply with the study plan, or have a study-related injury or for any other reason, the study physician may terminate your continued participation in this study.

You can be aware of the information and progress of the study, if you have questions related to this study, or if you have any discomfort or injury during the study, or have questions about the interests of participants in this study_______*(Phone number) with* Li CAI *(Name of the investigator or the relevant person) Contact.*

Informed consent form signing page

I have read this informed consent form.

I have the opportunity to ask questions and all the questions have been answered.

I understand that participation in this study is voluntary.

I may choose not to participate in this study or withdraw at any time without discrimination or retaliation, and any of my medical treatment and interests will not be affected.

If I need other treatment, or if I fail to comply with the study plan, or have a study-related injury or for any other reason, the study physician may terminate my continued participation in this study.

I will receive a signed copy of the "informed consent form".

Subjects signed:___________________Subject Contact Number:_______

date:______year_______moon_______sun

Signature of the legal agent: the relationship with the subject:_

contact number:____________________

date:______year_______moon_______sun

Guardian Signature: Relationship with the Subject:_______________

contact number:____________________

date:______year_______moon_______sun

I confirm that the details of the study including its rights and possible benefits and risks were explained to the subject and I give a copy of the signed informed consent form.

Investigator's Name:_________________

Investigator's signature:________________

date:_______year________moon________sun

(Note: If the subject is illiterate and fashionable, the witness needs the signature, and if the subject is incompetent, the agent or guardian needs the signature)

Office of Biomedical Research Ethics Committee, Affiliated Hospital of Zunyi Medical University Tel.: 0851-28608776
